# Central-to-Folding Chirality Control: Asymmetric Synthesis of Multilayer 3D Targets With Electron-Deficient Bridges

**DOI:** 10.3389/fchem.2022.860398

**Published:** 2022-03-31

**Authors:** Shengzhou Jin, Jia-Ying Wang, Yao Tang, Hossein Rouh, Sai Zhang, Ting Xu, Yu Wang, Qingkai Yuan, Daixiang Chen, Daniel Unruh, Guigen Li

**Affiliations:** ^1^ School of Chemistry and Chemical Engineering, Institute of Chemistry and BioMedical Sciences, Nanjing University, Nanjing, China; ^2^ Department of Chemistry and Biochemistry, Texas Tech University, Lubbock, TX, United States; ^3^ Continuous Flow Engineering Laboratory of National Petroleum and Chemical Industry, Changzhou University, Changzhou, China

**Keywords:** multilayer folding chirality, asymmetric synthesis, asymmetric Suzuki–Miyaura coupling, central-to-folding chirality, planar packing

## Abstract

New multilayer 3D chiral molecules have been designed and synthesized asymmetrically through the strategy of center-to-multilayer folding chirality control and double Suzuki couplings. Individual diastereoisomers were readily obtained and separated *via* flash column chromatography. The key diastereoisomer was further converted into corresponding enantiomers. These enantiomers possess electron-deficient aromatic bridges layered with top and bottom aromatic scaffolds. X-ray structural analysis has unambiguously confirmed the configuration, and intermolecular packing results in regular planar patterns in solid crystals. The synthesis was achieved in a total of ten steps starting from commercially available starting materials.

## Introduction

Chiral domains widely exist in natural and biological products and have been utilized in chiral catalysts and ligands for asymmetric transformations (Corey et al., 2012; Ito and Nozaki, 2010; Taniguchi et al., 2011; Smith and Jones, 2008). Asymmetric synthesis of both natural and unnatural enantiopure compounds through chiral controllers has been actively pursued for more than half a century (Ito and Nozaki, 2010). This topic will continue to be prevalent in academic laboratories and pharmaceutical and materials industries (Smith and Jones, 2008). Among asymmetric methodologies, those protocols by taking advantage of planar and helical chirality including corresponding chiral *π*-stacked scaffolds are particularly attractive to chemical and materials communities and have been well documented in the literature (Chen and Shen, 2017; Stará and stary, 2020; Anger et al., 2012). For example, chiral ferrocene ligands, which feature a unique sandwich-like structure, have been implemented in asymmetric catalysis (Dai et al., 2003; Arrayás et al., 2006). Double-layered enantiopure [2.2]paracyclophanes have also been employed as chiral ligands for transition metal–catalyzed asymmetric reactions (Mukhopadhyay et al., 2012; Spuling et al., 2018). Similarly, chiral *π*-layered [n]helicene derivatives have been employed for materials research regarding polarized organic electronics, optoelectronic chiroptical properties, etc. (Chen and Shen, 2017). In biological systems, DNA is one of the most instrumental biomolecules notably featuring multilayer paired chirality (Moser and Dervan, 1987; Gong et al., 2018; Knouse et al., 2018). The design and synthesis of chiral targets with novel multilayer structures for chemical and biological research have still been a significant and ongoing challenge.

**FIGURE 1:**
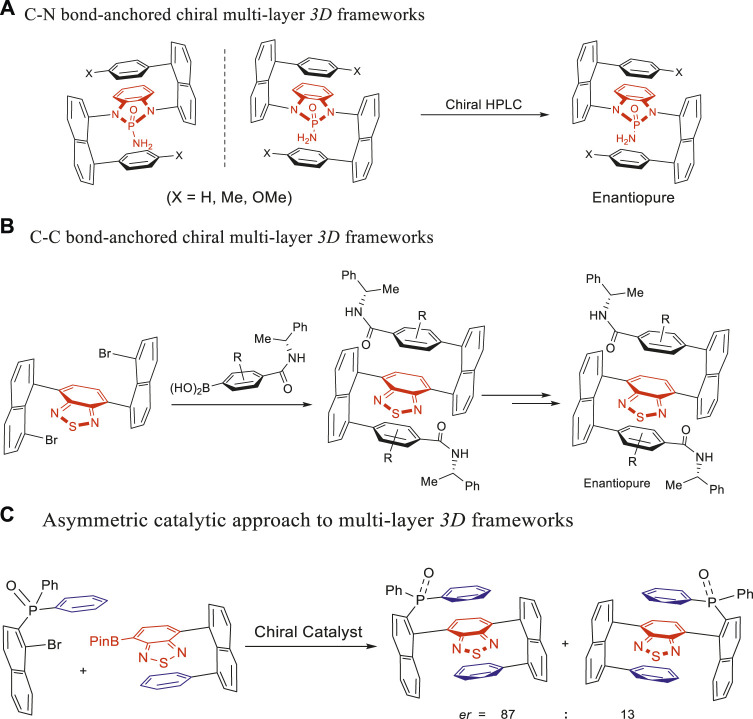
Strategy and progress of assembling multilayer 3D chirality. **(A)** C–N bond–anchored chiral multilayer 3D frameworks. **(B)** C–C bond–anchored chiral multilayer 3D frameworks. **(C)** Asymmetric catalytic approach to multilayer 3D frameworks.

Recently, our group has reported multilayer 3D folding chirality (Wu et al., 2019; Liu et al., 2020; Wu et al., 2020; Zhang and kurti, 2021; Wu et al., 2020) allowing pushing–releasing flexibility of structures, which is different from the rigid layered chirality documented in the literature. This multilayer folding chirality was inspired by our GAP chemistry and technology (Wu et al., 2020; Zhang and kurti, 2021). Figure 1A depicts the C–N bond–based multilayer targets prepared via double Buchwald–Hartwig cross-couplings (Wu et al., 2019; Liu et al., 2020) and featuring pseudo-*C*_*2*_-symmetry. Figure 1B illustrates the C–C bond–based multilayer 3D chiral counterparts, prepared via asymmetric double Suzuki–Miyaura cross-couplings (Wu et al., 2020; Wu et al., 2020; Zhang and kurti, 2021). Interestingly, when the aforementioned chiral compounds were irradiated with 365 nm UV light, various colors in solutions and macro-chirality patterns in solid states were witnessed with the naked eyes without the aid of a scanning device (Wu et al., 2020). Chiral separation via chiral HPLC and chiral auxiliary–based method was conducted for multilayer targets presented in Figure 1A and Figure 1B, respectively.

Asymmetric catalysis using chiral amide–phosphine ligands resulted in another category of C–C bond–based multilayer 3D chiral targets as shown in Figure 1C (Wu et al., 2020). These compounds would demonstrate new potentials as chiral phosphine ligands for transition metal–mediated reactions upon removing oxygen from the P=O group. Interestingly, these targets presented a new chiral framework containing a pseudo- or pro-chiral center and orientational or rotational axis. The pseudo-chiral center on the phosphorus atom was attached by one naphthalenyl ring and two identical aryl groups but differentiated by planar packing. This pseudo- or pro-chiral center can be extended to other centers of the tetrahedron (e.g., C and Si) or higher polyhedrons in the future. Obviously, atropisomers’ operation along the C–P bond axis made the orientation chirality possible. Three potential rotamers are generated by three moieties of diarylphosphine oxide scaffolds (two differentiated aromatic rings and one P=O group).

We also designed and assembled triple-columned and multiple-layered chiral folding polymers via asymmetric catalytic polymerization based on the use of various new monomers (Tang et al., 2022; Wang et al., 2022). The resulting chiral folding polymers exhibited remarkable optical properties in aggregated states (photoluminescence in solids and aggregation-induced emission in solutions), as well as reversible redox properties in electrochemical performance.

**SCHEME 1:**
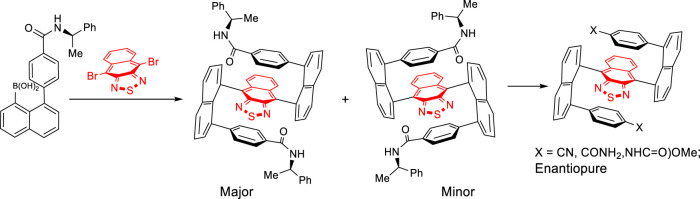
Asymmetric synthesis of multilayer chiral folding molecules.

In this work, we would like to report the design and synthesis of enantiomers possessing multilayer 3D folding chirality by utilizing a bridge of electron-deficient scaffolds. The extended central bridge layer can completely avoid possible racemization via rotational operations (Scheme 1) due to the extended aromatic system. The synthetic strategy of central-to-multilayer folding chirality and double Suzuki couplings was proven effective in stereocontrol and chemical yields.

## Results and Discussion

**SCHEME 2:**
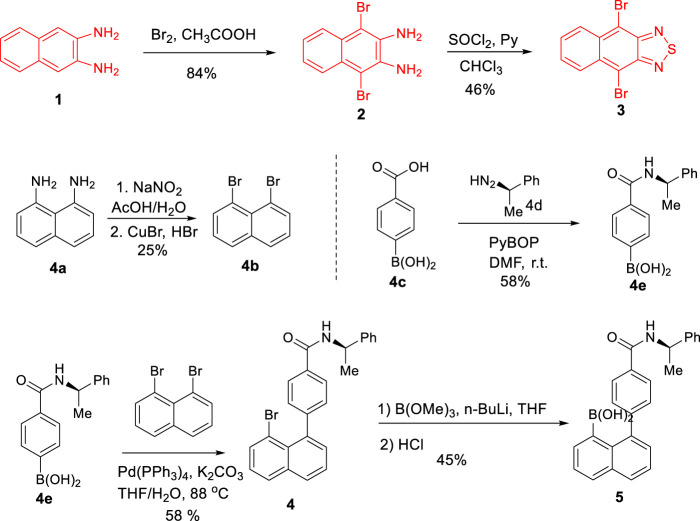
Synthesis of building blocks of **3** and **5**.

We started this project by synthesizing 4,9-dibromo-2,1,3-naphthothiadiazole (**3**) at first (Scheme 2). As shown in Scheme 1, 3 was obtained by treating 2,3-diaminonaphthalene (**1**) with bromine in acetic acid at room temperature followed by intramolecular cyclization with thionyl chloride in mixed solvents of pyridine and chloroform. The next step was furnished by stirring the reaction mixture at room temperature for 2 h and then refluxing for 8 h to give compound **3** in a two-step yield of 39% (Wu et al., 2020; Wu et al., 2021).

1,8-Dibromonaphthalene (**4b**) was generated through oxidative cyclization by treating naphthalene-1,8-diamine (**4a**) with sodium nitrite in an aqueous solution in the presence of acetic acid to afford 1H-naphtho[1,8-de][1,2,3]triazine (Beletskaya et al., 2007). The resulting hetero five-membered ring of triazine was opened by continuously treating with sodium nitrite and then with a mixture of CuBr in 47% HBr solution to give **4b** in a yield of 25% (Noland et al., 2011).

Precursor **4e**, (*R*)-(4-((1-phenylethyl)carbamoyl)phenyl)boronic acid, was obtained according to a classical procedure (Coste et al., 1990). In this preparation, 4-carboxybenzeneboronic acid (**4c**) (1.0 equiv) was treated with PyBOP (2.0 equiv) in DMF stirring for a few minutes, followed by adding (*R*)-(1-phenylethyl)diazene (2.0 equiv) into the reaction mixture. The carbonyl coupling was completed within 14 h at room temperature before quenching. Work-up and purification *via* column chromatography gave **4e** in a chemical yield of 58%.

Precursor **4**, (*R*)-4-(8-bromonaphthalen-1-yl)-N-(1-phenylethyl)benzamide, was prepared by performing Suzuki coupling of (*R*)-(4-((1-phenylethyl)carbamoyl)phenyl)boronic acid (**4e**) with 1,8-dibromonaphthalene (**4b**) under the standard catalytic condition with a moderate yield of 58%. (*R*)-(8-(4-((1-Phenylethyl)carbamoyl)phenyl)naphthalen-1-yl)boronic acid (**5**) was obtained by treating (*R*)-4-(8-bromonaphthalen-1-yl)-N-(1-phenylethyl)benzamide **4** with n-BuLi followed by the reaction with B(OMe)_3_ under Ar protection (Ishihara et al., 2002). After aqueous HCl hydrolyzed the resulting boronic ester, the precursor **5** was obtained via flash silica gel chromatography in 45% yield as a yellow powder solid.

For the synthesis of diastereoisomers, **6a** and **6b**, double Suzuki couplings turned out to be the crucial step for asymmetrically constructing the two C–C bonds (Wu et al., 2020). Investigations on the optimization of the coupling reaction of **3** with **5** became necessary. At first, the reaction was conducted using Pd(PPh_3_)_4_ as the catalyst and K_2_CO_3_ as the base in THF/H_2_O at 90°C for 36 h under argon protection (Table 1, entry 1). Surprisingly, almost no product was detected under this condition. To further optimize the reaction, various conditions, such as toluene/H_2_O, Et_2_O/H_2_O, and 1,4-dioxane/H_2_O as the solvent and K_2_CO_3_ as the base (Table 1, entries 2–4) and toluene as the solvent and K_3_PO_4_ as the base (Table 1, entry 5), were examined. These changes did not show satisfactory improvements either. To our delight, when Pd(OAc)_2_/PPh_3_ was employed as the catalyst, and xylene/H_2_O as the solvent at 130°C for 36 h, the yield was improved to 32% (Table 1, entry 6). Subsequently, as the reaction time was extended to 60 h, the yield increased to 45% (Table 1, entry 7).

**TABLE 1:** Optimization of the reaction conditions for the palladium-catalyzed Suzuki coupling of **3** with **5**.

	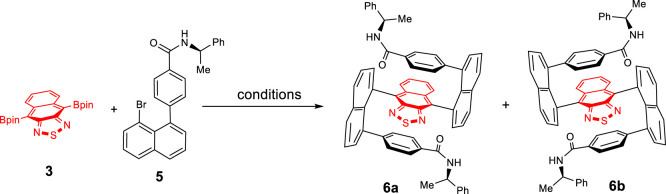	
Entry	Conditions^a^	Yield^b^
1	**3** (1 equiv), **5** (3 equiv), Pd(PPh_3_)_4_ (0.2 equiv), K_2_CO_3_ (6 equiv), THF/H_2_O (5:1, *v*/*v*), 36 h, 90°C	Trace
2	**3** (1 equiv), **5** (3 equiv), Pd(PPh_3_)_4_ (0.2 equiv), K_2_CO_3_ (6 equiv), PhMe/H_2_O (5:1, *v*/*v*), 36 h, 90°C	Trace
3	**3** (1 equiv), **5** (3 equiv), Pd(PPh_3_)_4_ (0.2 equiv), K_2_CO_3_ (6 equiv), Et_2_O/H_2_O (5:1, *v*/*v*), 36 h, 90°C	Trace
4	**3** (1 equiv), **5** (3 equiv), Pd(PPh_3_)_4_ (0.2 equiv), K_2_CO_3_ (6 equiv), 1,4-dioxane/H_2_O (5:1, *v*/*v*), 36 h, 90°C	-
5	**3** (1 equiv), **5** (3 equiv), Pd(PPh_3_)_4_ (0.2 equiv), K_3_PO_4_ (6 equiv), PhMe, 36 h, 120°C	Trace
6	**3** (1 equiv), **5** (3 equiv), Pd(OAc)_2_ (0.2 equiv), PPh_3_ (0.6 equiv), K_2_CO_3_ (6 equiv), xylene/H_2_O (5:1, *v*/*v*), 36 h, 130°C	32%
7	**3** (1 equiv), **5** (3 equiv), Pd(OAc)_2_ (0.2 equiv), PPh_3_ (0.6 equiv), K_2_CO_3_ (6 equiv), xylene/H_2_O (5:1, *v*/*v*), 60 h, 130°C	45%

a Reaction concentration conducted on **3** (0.1 mmol).

b Isolated yield after purification by silica gel chromatography based on **3**.

**SCHEME 3:**
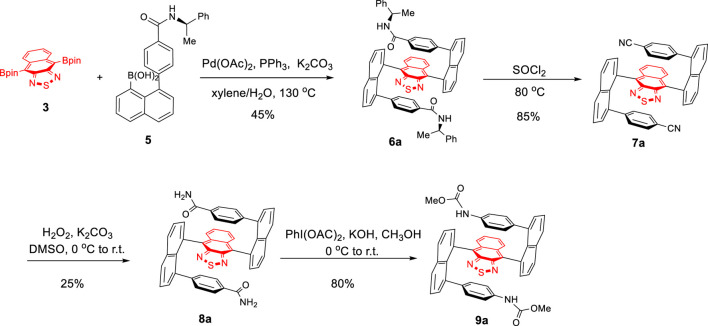
Asymmetric synthesis of multilayer 3D target **9a**.

As shown in Scheme 3, the treatment of **6a** with thionyl chloride at 80°C for 8 h afforded **7a** in 85% yield (Lv et al., 2016). At room temperature, oxidative hydrolysis of **7a** by H_2_O_2_ and K_2_CO_3_ gave **8a** in 25% yield (Mothana et al., 2010). The yield was improved to 55% after the unreacted starting material **6a** was recovered. Hofmann rearrangement of **7a** by PhI(OAc)_2_ and KOH in CH_3_OH afforded **9a** in 80% yield (Zhang et al., 2016).

## X-Ray Structure and Intermolecular Packing

**FIGURE 2:**
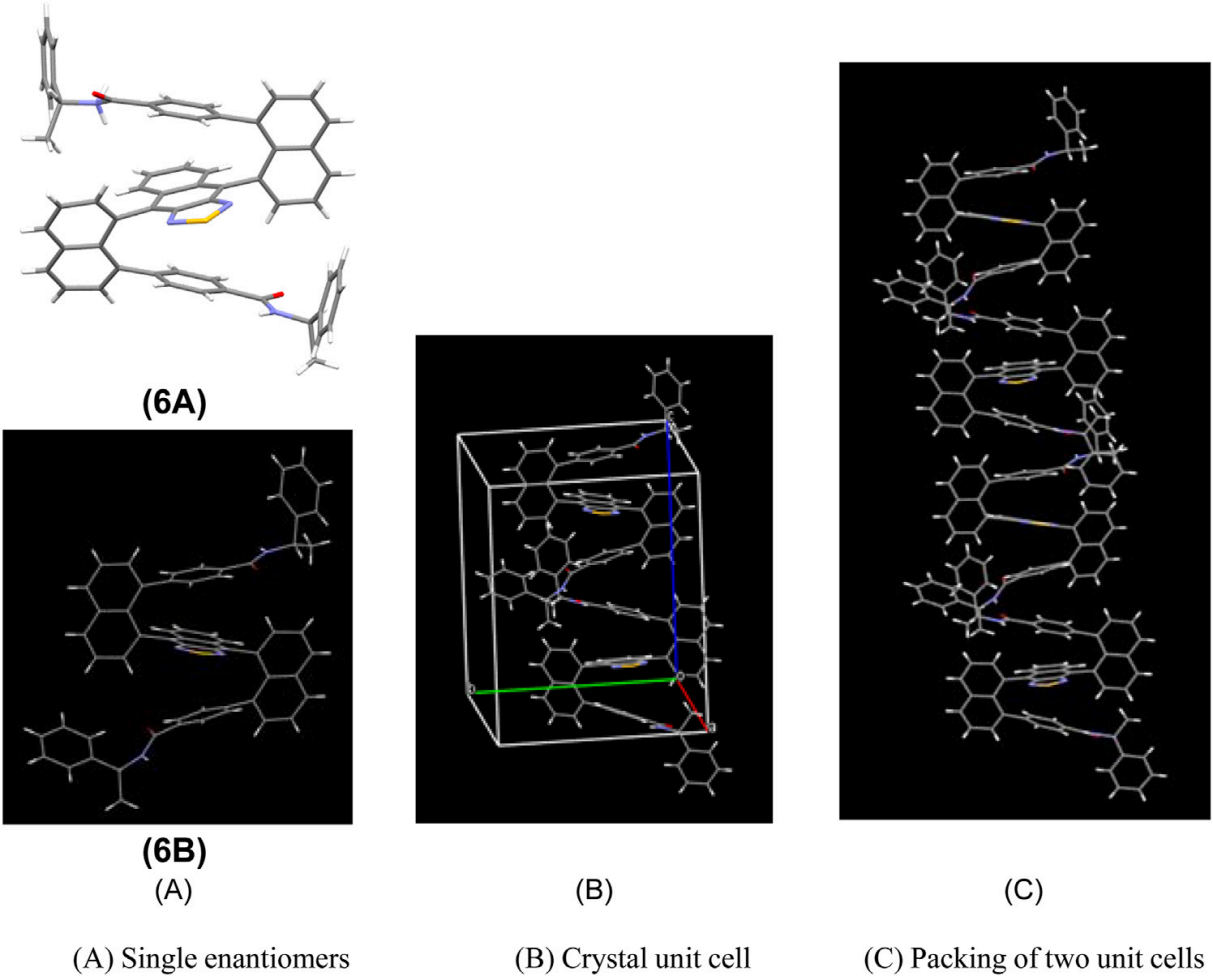
Single-crystal structure of **6a** and **6b** and intermolecular packing. **(A)** Single enantiomers. **(B)** Crystal unit cell. **(C)** Packing of two unit cells.

Product **6** was obtained as a red solid showing the diastereoselectivity of two diastereoisomers, **6a–6b**, as 1.6:1 dr. Two isomers were readily separated from the silica gel column. Single crystals of both **6a** and **6b** were grown by slowly diffusing hexane into the dichloromethane solution. X-ray crystal structure analysis confirmed the configurations of **6a** and **6b** (Supplementary Material and Figure 2A). This structure shows the two naphthalene columns are nearly perpendicular to the central naphthothiadiazole ring. This structural arrangement is slightly different from that of a previously bicyclic bridge-based counterpart in which two naphthalene piers are positioned at a dihedral angle of about 60^°^ (Wu et al., 2020). The up and down planes of the present molecules are almost parallel to the central layer in a similar arrangement to the aforementioned bicyclic bridge-based structural system (Figure 2A). The enantiomers’ packing in a single X-ray unit cell represents an interesting arrangement in which the intermolecular distances between proximate aromatic rings are very similar to intramolecular distances (Figure 2B). Additionally, the phenyl ring of one of the amide groups is oriented nearly parallel to the naphthalene piers on two sides. Interestingly, the packing between two crystal unit cells displays a similar distance to that inside a single unit cell. This pattern involves 12 layers in one packing column (Figure 2C).

## UV–Vis and Fluorescence Studies

**FIGURE 3:**
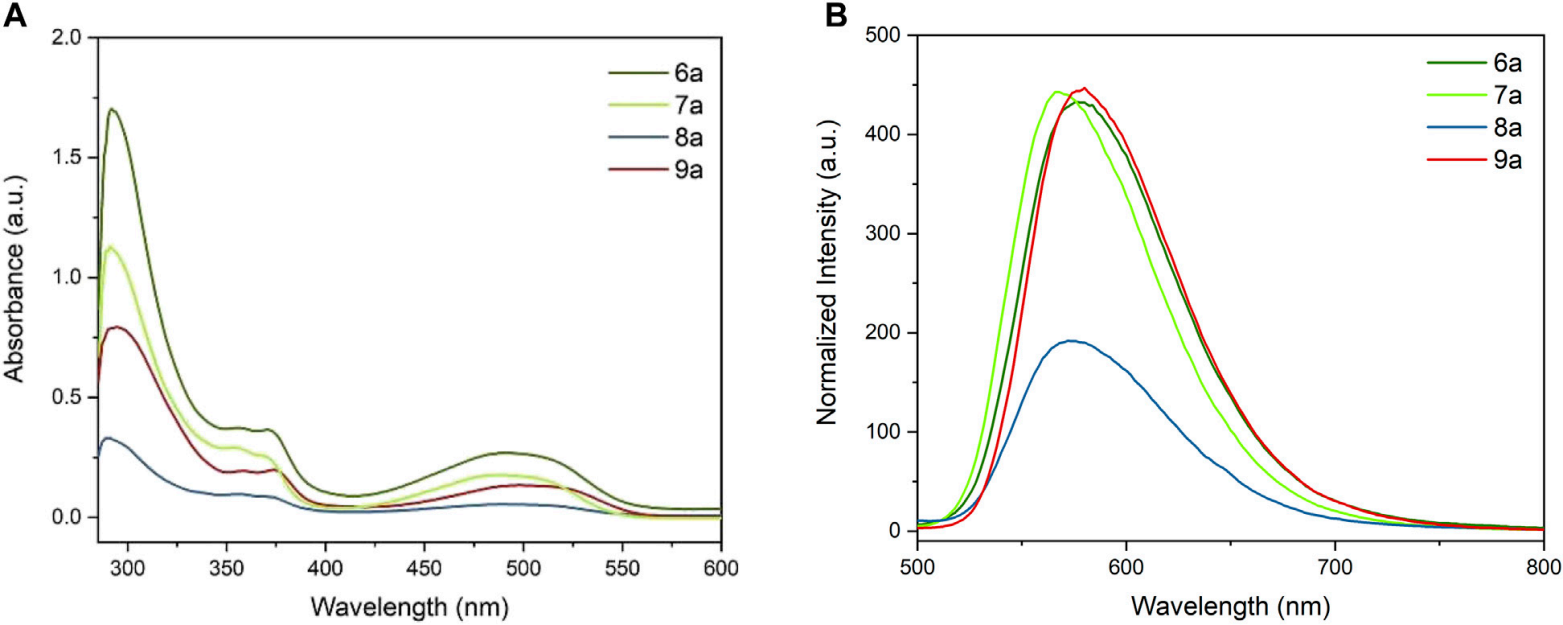
**(A)** UV–Vis absorbance of **6a–9a** (0.05 mg/ml) in THF. **(B)** Normalized fluorescent spectra of **6a–9a** (0.05 mg/ml) in THF. Excitation wavelength for **6a–9a**: 323 nm.

As shown in Figure 3A, **6a**–**9a** exhibited a similar spectral behavior, featuring a sharp absorption band between 285 and 350 nm and one broader band between 420 nm and 560 nm. In **6a**, **8a**, and **9a**, the π–π conjugation between the aromatic rings and the adjacent carbonyl groups leads to an extended conjugated system. A significant red shift was observed in **9a**, which moved to a longer wavelength by about 15 nm than **6a**. It is worth noting that the peak intensity of **8a** was sharply lower than the intensity of other derivatives.

The fluorescence emission studies of **6a**–**9a** were performed in THF by performing excitation at the same wavelength, and all derivatives displayed a broad emission band around 520–740 nm (Figure 3B). Both **6a** and **9a** showed a strong fluorescence emission at about 576 nm. The nitrile group with a stronger electron-withdrawing effect made the maximum fluorescent emission peaks shift to a shorter wavelength (568 nm, compared to **6a** and **9a**). Like the absorption spectrum, the emission intensity of **8a** was diminished mainly, which was half of that of the other derivatives. Based on the spectral observation on absorption and emission, it can be concluded that different electron-withdrawing groups influence the conjugating system in various manners, resulting in an increase/decrease in π–π* energy gaps. The different effects on conjugations are probably one of the reasons for the shift of absorption to higher wavelengths and lowered emission; however, the coordination of those groups with the solvent could also contribute to the observed change in both spectrums.

### 1,4-Dibromonaphthalene-2,3-diamine (**2**)

A mixture of 3.45 ml of bromine (20.7 g, 85.5 mmol) and 90 ml of glacial acetic acid was added dropwise into a solution of 2,3-diaminonaphthalene (5.0 g, 31.6 mmol) in 140 ml of glacial acetic acid, with vigorous stirring at room temperature. After 8 h, water was added to the solution. The precipitate was filtered off and washed subsequently with glacial acetic acid and water. After drying, a brown powder (8.4 g, 84%) was obtained.

### 4,9-Dibromonaphtho[2,3-c][1,2,5]thiadiazole (**3**)

A solution of **2** (8.1 g, 25.6 mmol) in 280 ml of chloroform was added dropwise into the mixture of thionyl chloride (12.7 ml, 20.8 g, 174.8 mmol) in 280 ml of chloroform and 31 ml of pyridine, with vigorous stirring in an ice-water bath. After the dropwise addition, the solution was stirred for 2 h at room temperature and refluxed overnight. Evaporation of the solvent and purification by column chromatography on silica gel with dichloromethane–hexane as the eluent were performed, and an orange-color product (4.5 g, 46%) was obtained.

### (R)-4-(8-Bromonaphthalen-1-yl)-N-(1-phenylethyl)benzamide (**4**)

**4e** (3.7 g, 13.9 mmol), **4b** (4.0 g, 13.9 mmol), Pd(PPh_3_)_4_ (0.8 g, 0.7 mmol), and anhydrous K_2_CO_3_ (5.8 g, 42.0 mmol) were dissolved into THF/H_2_O (100 ml/20 ml), and the solution mixture was degassed and charged with argon. The solution was heated to 88°C and stirred overnight. After the reaction was completed, the resulting mixture was evaporated, and the residue was diluted with EtOAc. The organic phase was washed twice with water and brine, dried over Na_2_SO_4_, and concentrated under reduced pressure. Work-up via column chromatography (DCM: acetone = 10/1) gave **4** as a white solid (3.5 g, 58% yield).

### (*R*)-(8-(4-((1-Phenylethyl)carbamoyl)phenyl)naphthalen-1-yl)boronic Acid (**5**)

In a dried and argon-flushed round bottom flask with a stir bar, bromide substrate **4** (10 mmol) was dissolved into anhydrous THF and stirred for 5 min at −78°C. 1.6 M n-butyllithium (25 mmol) solution was added dropwise with syringe and stirred at −78°C for 0.5 h. Then, B(OMe)_3_ (40 mmol) was added dropwise at −78°C; the reaction mixture was warmed up to RT and stirred for 8 h. Then, 1 M HCl (10 mmol) was added, and the mix was stirred for 6 h. After monitoring by TLC analysis, extraction with EA, and drying for column (Hexane/EA = 5/1 to 1/1), we obtained the crude solid, which can be directly used in the next step.

### 4,4′-(Naphtho[2,3-c][1,2,5]thiadiazole-4,9-diylbis(naphthalene-8,1-diyl))bis(N-((*R*)-1-phenylethyl)benzamide) (**6a**)

**3** (60 mg, 1 equiv), chiral boronic acid **5** (200 mg, 3 equiv), Pd(OAc)_2_ (8 mg, 0.2 equiv), PPh_3_ (14 mg, 0.6 equiv), and K_2_CO_3_ (73 mg, 6 equiv) were dissolved into xylene/H_2_O (5 ml/1 ml), and the solution mixture was degassed with argon. The resulting solution was heated to 130°C and stirred for 60 h. Work-up via column chromatography (DCM/acetone, 20/1) afforded the pure isomer **6** as a red solid (70 mg, 45% yield).

### 4,4′-(Naphtho[2,3-c][1,2,5]thiadiazole-4,9-diylbis(naphthalene-8,1-diyl))dibenzonitrile (**7a**)

**6** (100 mg) was dissolved into CHCl_3_ (8 ml), SOCl_2_ (5 ml), and DMF (one drop). The resulting solution was heated to 80°C for 8 h. The reaction was monitored by TLC analysis and worked up for column (DCM/acetone = 20/1) to afford the pure product **7** as a red solid (62 mg, 85% yield).

### 4,4′-(Naphtho[2,3-c][1,2,5]thiadiazole-4,9-diylbis(naphthalene-8,1-diyl))dibenzamide (**8a**)

To the solution of **7** (50 mg, 0.08 mmol) in DMSO (2 ml), cooled in an ice bath, were added 30% H_2_O_2_ (2 ml) and anhydrous K_2_CO_3_ (1.5 g, 11 mmol). The mixture was stirred and allowed to warm up to room temperature. After 30 min, distilled water (2 ml) and DMSO (2 ml) were added and cooled, and the mixture was stirred at room temperature for 18 h. The reaction mixture was diluted with EtOAc and quenched with saturated NH_4_Cl (2 ml). The organic phase was washed twice with water and brine, dried over Na_2_SO_4_, and concentrated under reduced pressure. Work-up via column chromatography (DCM:CH_3_OH = 5/1) afforded **8** as a red solid (13 mg, 25% yield).

### Dimethyl((naphtho[2,3-c][1,2,5]thiadiazole-4,9-diylbis(naphthalene-8,1-diyl))bis(4,1-phenylene))dicarbamate (**9a**)

To a solution of KOH (10 mg, 0.17 mmol) in methanol (5 ml) was added **8** (25 mg, 0.04 mmol). The mixture was stirred at room temperature until a homogeneous solution was obtained and then cooled to 0°C in an ice-water bath. Diacetoxyiodobenzene (23 mg, 0.07 mmol) was added in one portion. The mixture was stirred at ice bath temperature for 6 h. TLC was checked, the solvent was evaporated, and the column was run (DCM: acetone = 20: 1) to yield **9** as a red solid (9 mg, 80% yield).

## Conclusion

In conclusion, we have reported the design and asymmetric synthesis of new multilayer 3D chiral molecules through the strategy of central-to-multilayer folding chirality and double Suzuki couplings. Individual diastereopure isomers have been obtained and separated *via* flash column chromatography. The major key isomer has been converted into corresponding enantiomers. These enantiomers possess electron-deficient aromatic bridges and top and bottom layers. The absolute configuration of the products has been unambiguously determined by X-ray structural analysis. Intermolecular packing results in a regular planar pattern in crystals. The synthesis was achieved in a total of ten steps starting from commercially available starting materials.

## Data Availability

The original contributions presented in the study are included in the article/Supplementary Material, and further inquiries can be directed to the corresponding author.
