# Supplementary material for: Central-to-Folding Chirality Control: Asymmetric Synthesis of Multilayer 3D Targets With Electron-Deficient Bridges
Source: Front Chem. 2022 Mar 31;10:860398. doi: 10.3389/fchem.2022.860398 (PMC9010227; doi:10.3389/fchem.2022.860398)
Supplement: Supplementary file 1 [file DataSheet1.PDF]

Supplemental Materials

Central-to-Folding Chirality Control: Asymmetric Synthesis of Multi-Layer 3D Targets with Electron-Difficient Bridges

Shengzhou Jin,[+,1] Jia-Ying Wang,[+,1] Yao Tang,[+,2] Hossein Rouh,[2] Sai Zhang,[2] Ting Xu,[1] Yu Wang,[1] Qingkai Yuan,[2] Daixiang Chen,[3] Daniel Unruh[2] and Guigen Li*[,1,2]

[1]Institute of Chemistry and BioMedical Sciences, School of Chemistry and Chemical Engineering, Nanjing University, Nanjing, 210093, China;
[2]Department of Chemistry and Biochemistry, Texas Tech University, Lubbock, Texas 79409-1061, USA.
[3]Continuous Flow Engineering Laboratory of National Petroleum and Chemical Industry, Changzhou University，Changzhou, Jiangsu 213164, China.

[+] These authors contributed equally to this work.

Experimental section

1,4-dibromonaphthalene-2,3-diamine (2)

A mixture of 3.45 mL of bromine (20.7 g, 85.5 mmol) and 90 mL of glacial acetic acid was added dropwise into a solution of 2,3- diaminonaphthalene (5.0 g, 31.6 mmol) in 140 mL of glacial acetic acid, with vigorous stirring at room temperature. After 8 h, water was added into the solution. The precipitate was filtered off and washed subsequently with glacial acetic acid and water. After drying, a brown powder (8.4 g, 84%) was obtained. ^{1}H NMR (400 MHz, Chloroform-*d*) δ 7.99 (dt, *J* = 6.4, 3.2 Hz, 2H), 7.40 – 7.35 (m, 2H), 4.31 (s, 4H).

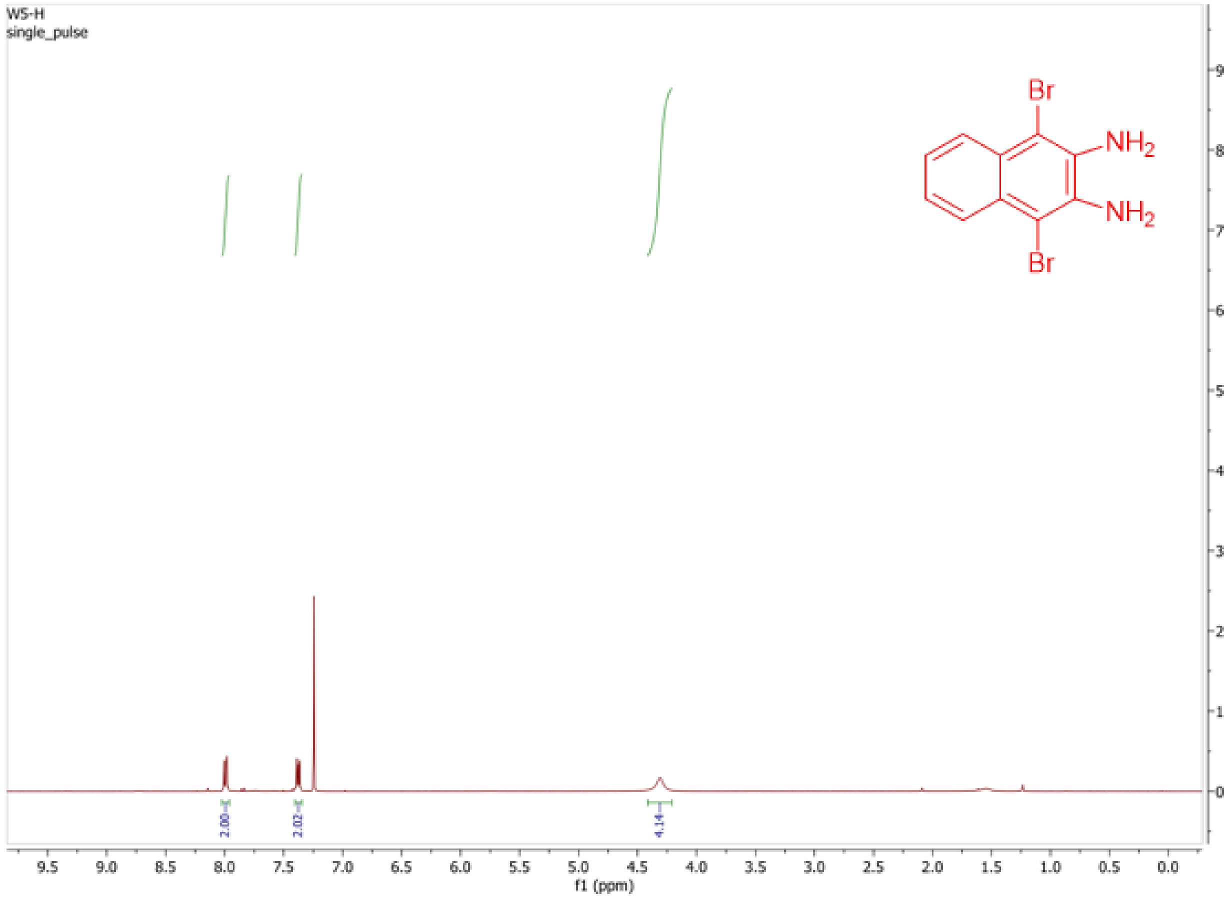

4,9-dibromonaphtho[2,3-c][1,2,5]thiadiazole (3)

A solution of **2** (8.1 g, 25.6 mmol) in 280 mL of chloroform was added dropwise into the mixture of thionyl chloride (12.7 mL, 20.8 g, 174.8 mmol) in 280 mL of chloroform, and 31 mL of pyridine, with vigorous stirring in an ice-water bath. After the dropwise addition, the solution was stirred for 2 h at room temperature, and refluxed overnight. Evaporation of the solvent and purification by column chromatography on silica gel with dichloromethane –hexane as the eluent were performed, and an orange-color product (4.5 g, 46%) was obtained. ^{1}H NMR (400 MHz, Chloroform-*d*) δ 8.41 (ddd, *J* = 8.4, 6.4, 3.3 Hz, 2H), 7.65 – 7.55 (m, 2H).

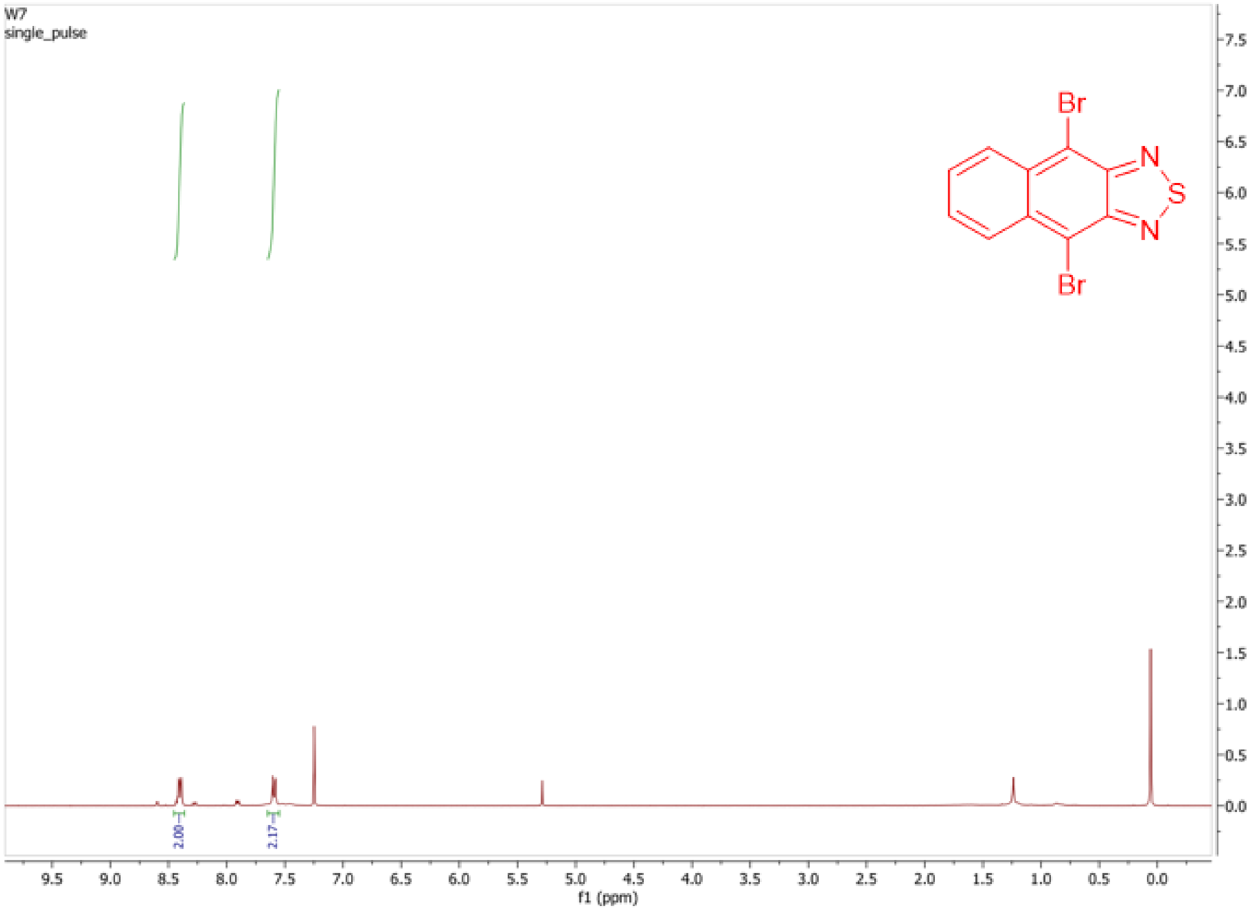

(*R*)-(8-(4-((1-phenylethyl)carbamoyl)phenyl)naphthalen-1-yl)boronic acid (5)
To a dried and argon-flushed round bottom flask with a stir bar, dissolved bromide substrate **4** (10 mmol) into anhydrous THF and stirred for 5 min at -78ºC. Transferred 1.6 M *n*-butyllithium (25 mmol) solution dropwise with syringe, stirred at -78ºC for 0.5 h. Then $B(OMe)_3$ (40 mmol) was added dropwise at -78ºC; reaction mixture was warmed up to r.t. and stirred for 8 h. Added 1 M HCl (10 mmol) and stirred the reaction for 6 h. Monitored by TLC analysis and exacted with EA, dried for column (Hexane/EA = 5/1 to 1/1) to afford the crude solid, which can be directly used in the next step.

4,4'-(naphtho[2,3-c][1,2,5]thiadiazole-4,9-diylbis(naphthalene-8,1-diyl))bis(N-((*R*)-1-phenylethyl)benzamide) (6a)
Dissolved **3** (60 mg, 1 eq), chiral boronic acid **5** (200 mg, 3 eq), $Pd(OAc)_2$ (8 mg, 0.2 eq), PPh_3 (14 mg, 0.6 eq), and K_2CO_3 (73 mg, 6 eq) into xylene/H_2O (5 mL/1 mL), degassed the solution mixture with argon. Heated the resulting solution to 130 °C and stirred for 60 h. Work up for column (DCM/acetone, 20/1) to afford the pure isomer **6** as a red solid (70 mg, 45% yield). ^1H NMR (400 MHz, Chloroform-*d*) δ 8.07 (ddd, *J* = 23.7, 8.3, 1.4 Hz, 4H), 7.72 – 7.64 (m, 2H), 7.50 (dd, *J* = 8.2, 7.0 Hz, 2H), 7.33 (dd, *J* = 7.0, 1.4 Hz, 2H), 7.20 (tt, *J* = 8.3, 3.8 Hz, 8H), 7.13 (dd, *J* = 6.8, 3.0 Hz, 4H), 7.02 (dd, *J* = 7.1, 1.4 Hz, 2H), 6.94 (dd, *J* = 7.0, 3.2 Hz, 2H), 6.61 (dd,

J = 7.9, 1.9 Hz, 2H), 6.51 (dd, *J* = 7.9, 1.9 Hz, 2H), 6.37 (dd, *J* = 7.9, 1.8 Hz, 2H), 6.13 (dd, *J* = 7.9, 1.8 Hz, 2H), 5.57 (d, *J* = 7.1 Hz, 2H), 5.08 (p, *J* = 6.8 Hz, 2H), 1.39 (d, *J* = 6.9 Hz, 6H). ^{13}C NMR (101 MHz, CHLOROFORM-*D*) δ 165.70, 151.18, 145.39, 143.10, 139.09, 134.97, 133.20, 132.21, 131.98, 131.42, 131.14, 130.65, 130.02, 129.80, 129.35, 128.70, 128.55, 127.49, 127.15, 126.72, 126.33, 125.99, 125.80, 125.11, 124.25, 122.95, 77.44, 77.12, 76.80, 49.39, 29.81, 21.70, 1.12. HRMS (ESI-TOF) m/z: $[M+H]^+$ calcd for $C_{60}H_{45}N_4O_2S$ 885.3263; Found 885.3313. $[\alpha]_D^{25}$ = 465.3 (*c* = 0.15, CH_2Cl_2).

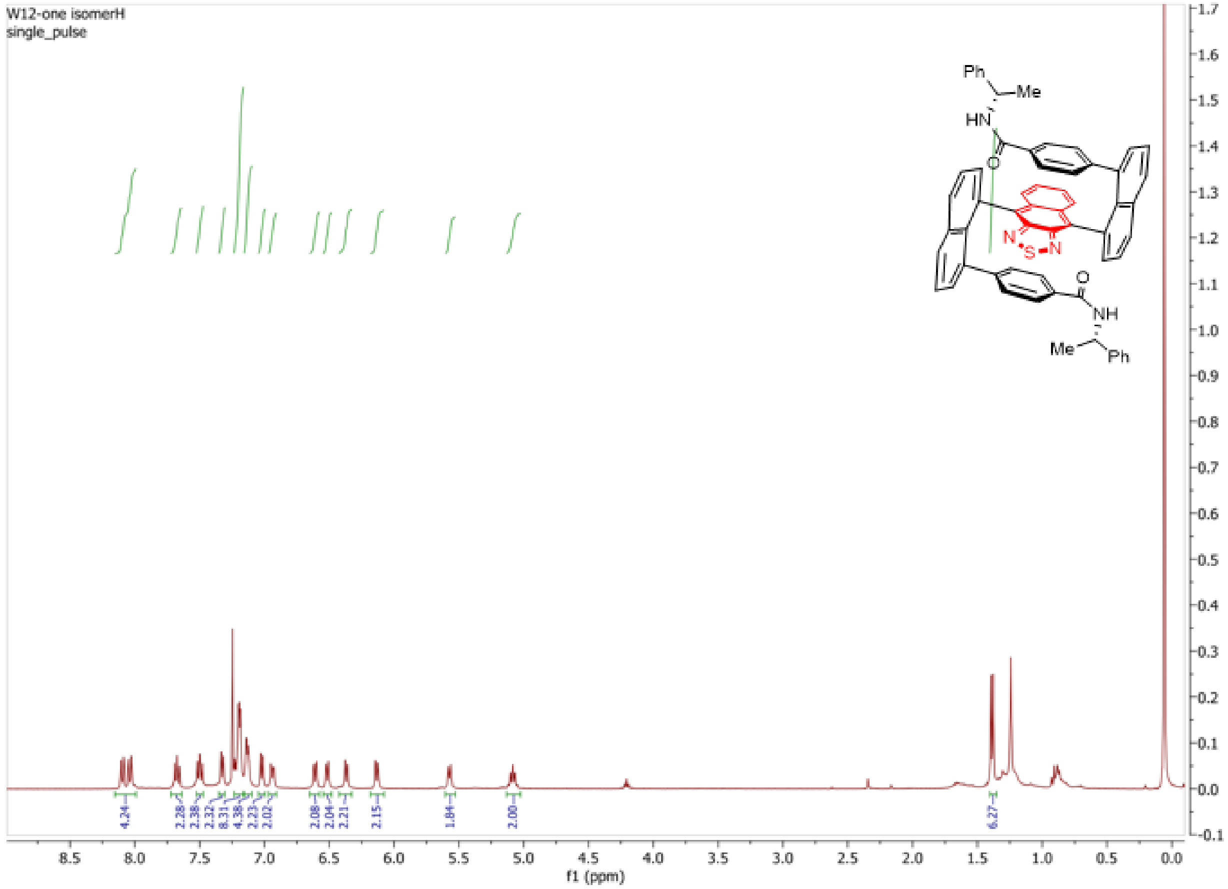

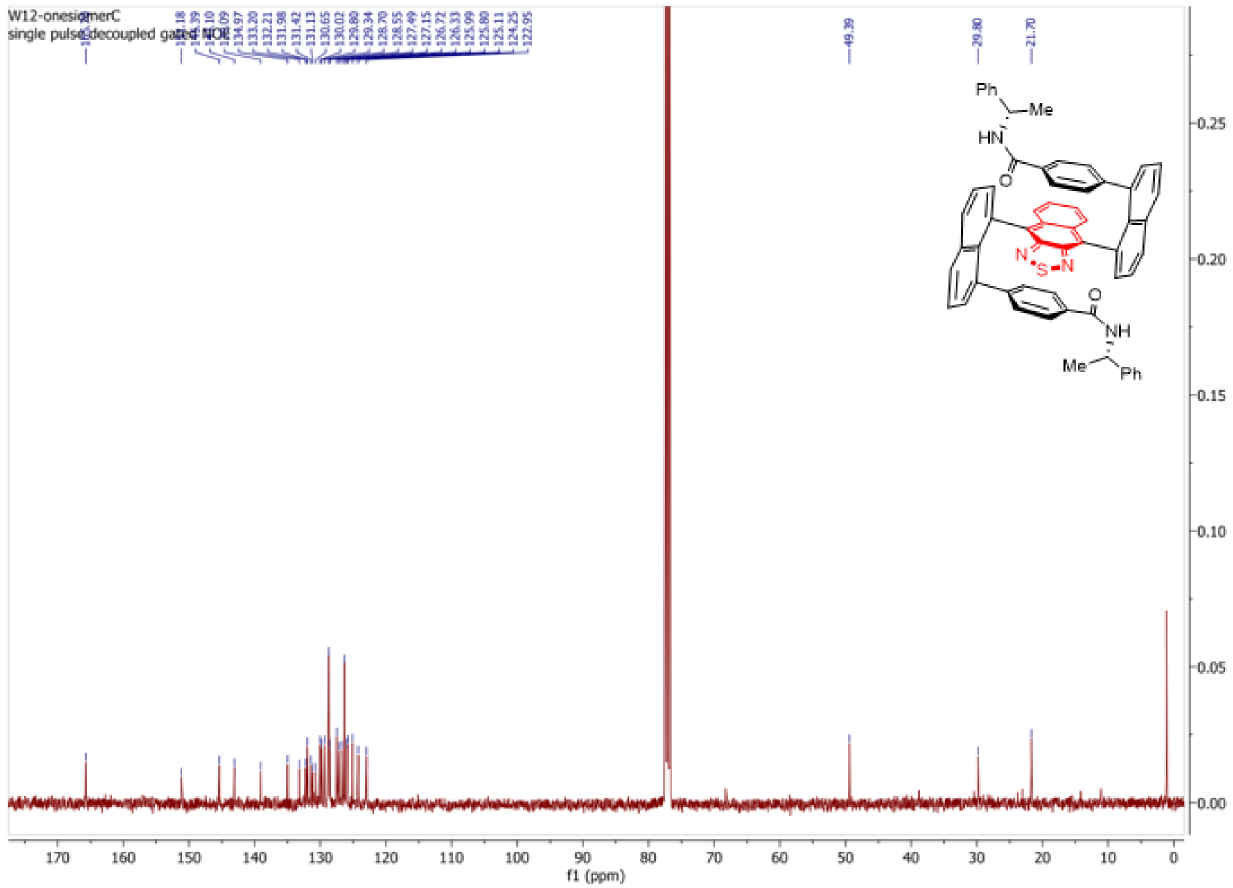

4,4'-(naphtho[2,3-c][1,2,5]thiadiazole-4,9-diylbis(naphthalene-8,1-diyl))dibenzonitrile (7a)
Dissolved **6** (100 mg) into $CHCl_3$ (8 mL), $SOCl_2$ (5 ml) and DMF (one drop). Heated the resulting solution to 80°C for 8 hours. Reaction was monitored by TLC analysis and worked up for column (DCM/acetone = 20/1) to afford the pure product **7** as a red solid (62 mg, 85% yield). ^{1}H NMR (400 MHz, Chloroform-*d*) δ 8.17 (dd, *J* = 8.3, 1.4 Hz, 2H), 8.08 (dd, *J* = 8.3, 1.4 Hz, 2H), 7.90 (dd, *J* = 8.2, 7.0 Hz, 2H), 7.52 (dd, *J* = 8.3, 7.0 Hz, 4H), 7.39 (dt, *J* = 6.2, 3.1 Hz, 2H), 7.19 (dd, *J* = 7.0, 3.2 Hz, 2H), 7.01 (dd, *J* = 7.0, 1.4 Hz, 2H), 6.48 (q, *J* = 1.3 Hz, 4H), 6.39 (dt, *J* = 7.9, 1.1 Hz, 2H), 6.25 (dt, *J* = 8.1, 1.2 Hz, 2H). ^{13}C NMR (101 MHz, CHLOROFORM-*D*) δ 191.95, 150.98, 146.98, 143.32, 139.77, 138.02, 137.00, 135.10, 132.58, 132.42, 132.10, 130.86, 130.52, 130.34, 130.15, 129.15, 128.74, 128.25, 127.52, 127.00, 126.63, 126.07, 125.17, 118.68, 109.02, 77.45, 77.13, 76.81, 29.80, 29.64, 15.77, 1.49, 1.12. $[M+H]^+$ calcd for $C_{44}H_{25}N_4S$ 641.1799; Found 641.1830 $[\alpha]_D^{25}$ = 172.0 (*c* = 1.0, CH_2Cl_2).

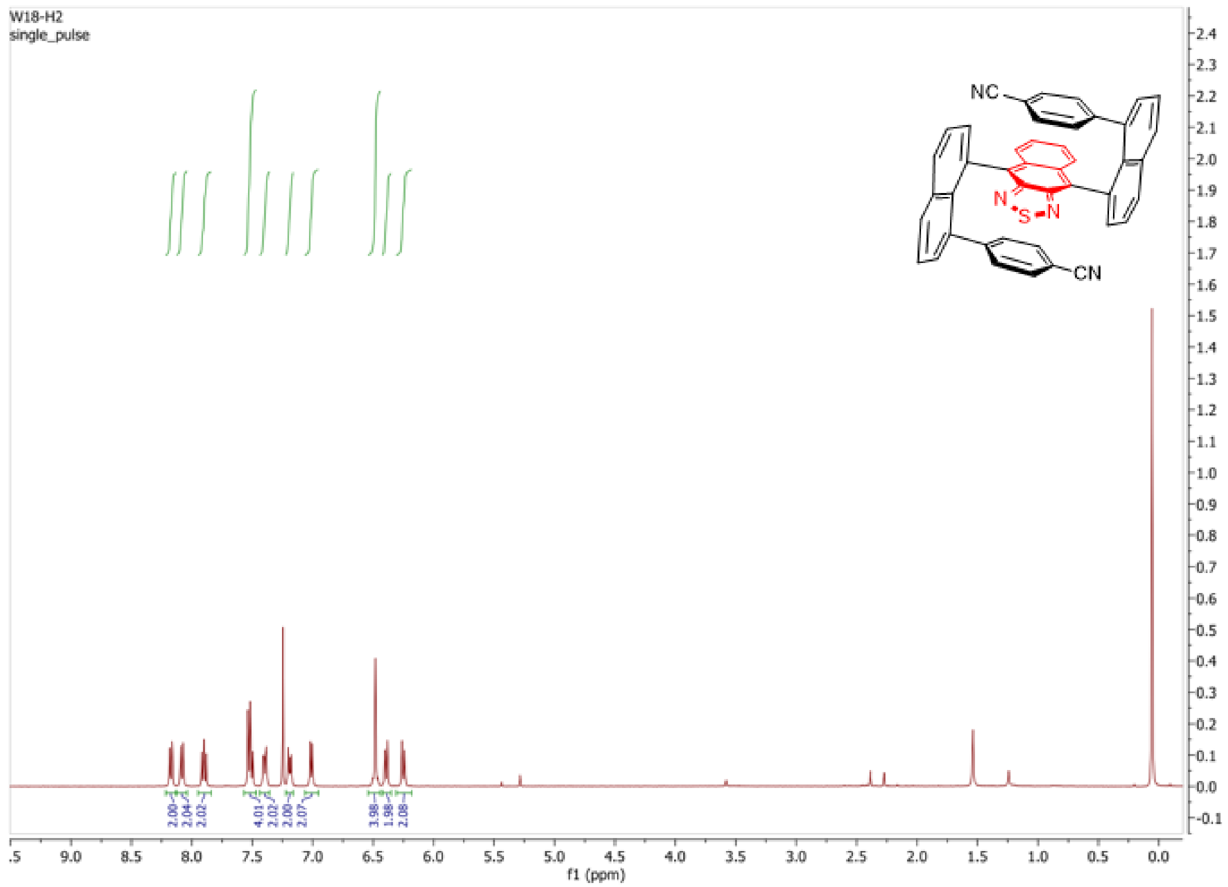
W18-H2
single_pulse
NC
N
S
N
CN
2.00
2.04
2.02
4.01
2.02
2.00
2.07
3.98
1.98
2.08
f1 (ppm)

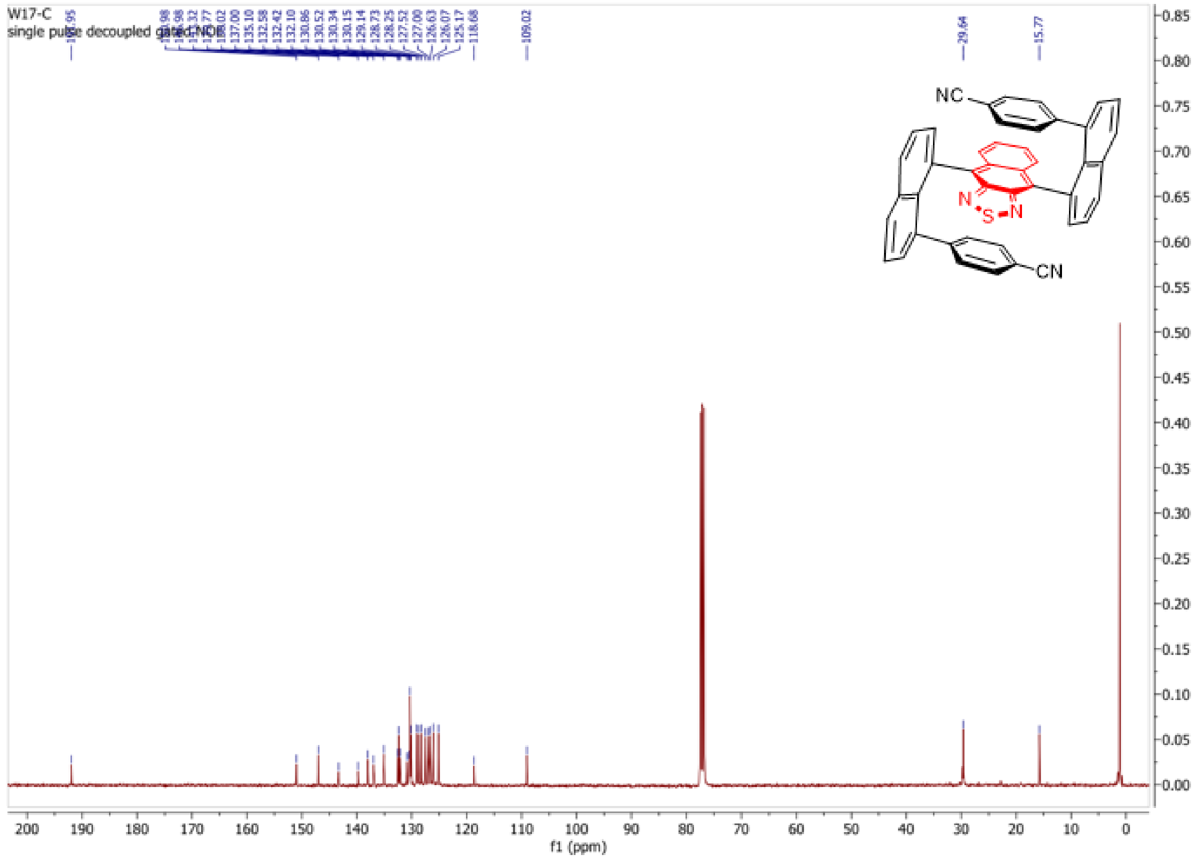

4,4'-(naphtho[2,3-c][1,2,5]thiadiazole-4,9-diylbis(naphthalene-8,1-diyl))dibenzamide (8a)

To the solution of **7** (50 mg, 0.08 mmol) in DMSO (2 mL), cooled in an ice bath, were added 30% H_2O_2 (2 mL) and anhydrous K_2CO_3 (1.5 g, 11 mmol). The mixture was stirred and allowed to warm up to room temperature. After 30 min, distilled water (2 mL) and DMSO (2 mL) were added, cooling applied, and the mixture was stirred at room temperature for 18 h. The reaction mixture was diluted with EtOAc and quenched with saturated NH_4Cl (2 mL). The organic phase was washed twice with water and brine, dried over Na_2SO_4, and concentrated under reduced pressure. Work up for column (DCM:CH_3OH = 5/1) to afford **8** as a red solid (13 mg, 25% yield). ^{1}H NMR (400 MHz, DMSO-d_6) δ 8.19 (dd, J = 8.4, 1.4 Hz, 2H), 8.11 (dd, J = 8.5, 1.3 Hz, 2H), 7.65 (dd, J = 8.2, 7.0 Hz, 2H), 7.54 – 7.48 (m, 4H), 7.25 – 7.20 (m, 6H), 7.16 (s, 2H), 6.99 (dd, J = 7.0, 1.4 Hz, 2H), 6.70 (dd, J = 7.9, 1.9 Hz, 2H), 6.65 (dd, J = 8.0, 1.9 Hz, 2H), 6.25 (dd, J = 8.0, 1.9 Hz, 2H), 6.06 (dd, J = 7.9, 1.9 Hz, 2H). ^{13}C NMR (101 MHz, DMSO-D_6) δ 167.24, 150.86, 144.81, 139.51, 135.06, 133.66, 132.57, 131.93, 131.20, 130.86, 130.56, 130.48, 129.89, 129.61, 127.84, 126.91, 126.64, 126.53, 125.83, 125.61, 124.96, 124.26, 55.85, 40.66, 40.45, 40.24, 40.03, 39.82, 39.61, 39.40, 29.52. $[M+H]^+$ calcd for $C_{44}H_{29}N_4O_2S$ 677.2011; Found 677.2048 $[\alpha]_D^{25}$ = 333.3 (c = 0.1, CH_2Cl_2).

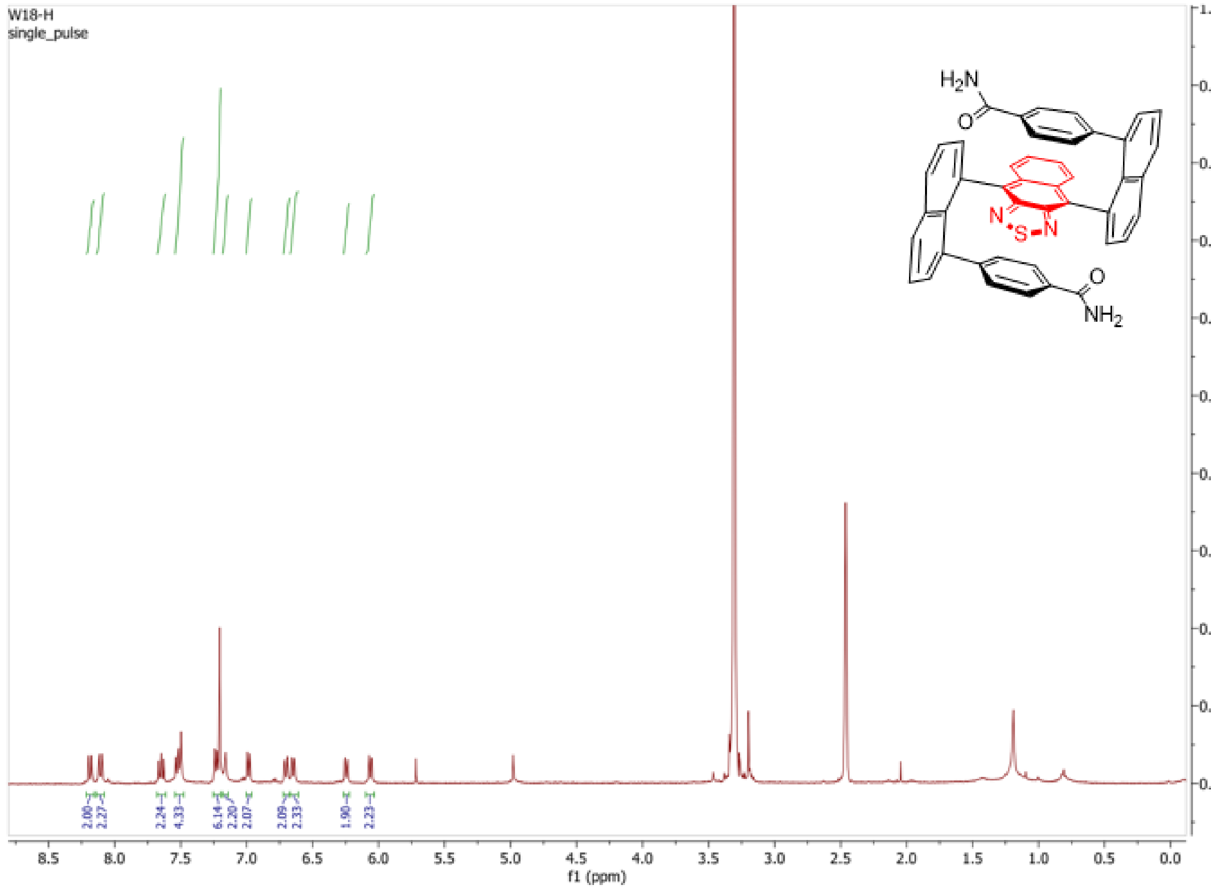
W18-H
single_pulse
H2N
O
N
S
N
O
NH2
2.00
2.27
2.24
4.33
6.14
2.20
2.07
2.09
2.33
1.90
2.23
8.5 8.0 7.5 7.0 6.5 6.0 5.5 5.0 4.5 4.0 3.5 3.0 2.5 2.0 1.5 1.0 0.5 0.0
f1 (ppm)
1.0 0.9 0.8 0.7 0.6 0.5 0.4 0.3 0.2 0.1 0.0

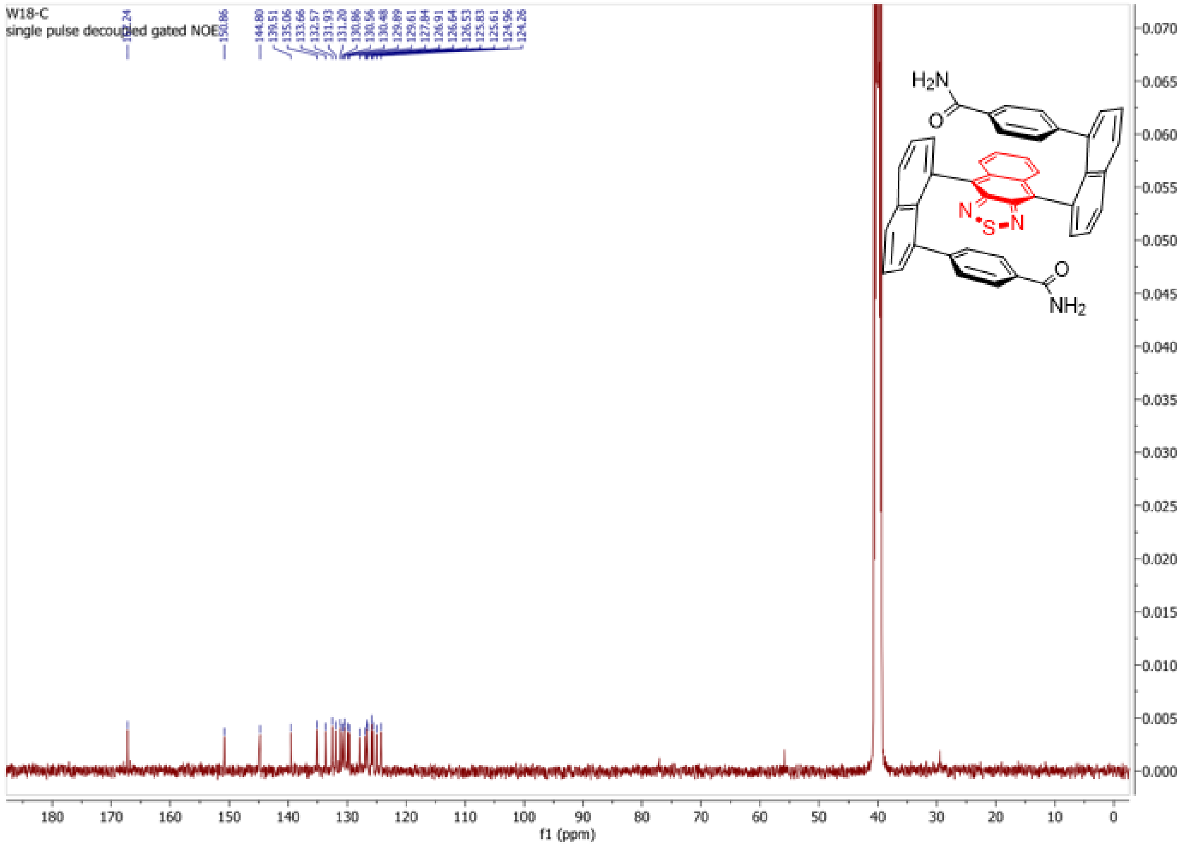

Dimethyl ((naphtho[2,3-c][1,2,5]thiadiazole-4,9-diylbis(naphthalene-8,1-diyl))bis(4,1-phenylene))dicarbamate (9a)

To a solution of KOH (10 mg, 0.17 mmol) in methanol (5 mL) was added **8** (25 mg, 0.04 mmol). The mixture was stirred at room temperature until a homogeneous solution was obtained, then cooled to 0 ^{o}C in an ice-water bath. Diacetoxyiodobenzene (23 mg, 0.07 mmol) was added in one portion. The mixture was stirred at ice bath temperature for 6 h. Check TLC. Evaporate the solvent and run the column (DCM: Acetone = 20 : 1) to yield **9** as a red solid (9 mg, 80% yield). ^{1}H NMR (400 MHz, Chloroform-*d*) δ 8.09 (dd, *J* = 8.3, 1.4 Hz, 2H), 8.00 (dd, *J* = 8.2, 1.3 Hz, 2H), 7.61 (t, *J* = 7.6 Hz, 2H), 7.51 – 7.44 (m, 2H), 7.33 (ddd, *J* = 20.5, 7.0, 2.3 Hz, 4H), 7.15 – 7.03 (m, 4H), 6.39 – 6.22 (m, 4H), 6.18 – 6.08 (m, 4H), 6.01 (d, *J* = 8.5 Hz, 2H), 3.76 (s, 6H). ^{13}C NMR (101 MHz, CHLOROFORM-*D*) δ 150.97, 139.73, 138.47, 137.28, 135.30, 135.08, 133.85, 132.30, 131.73, 131.42, 130.75, 130.49, 129.83, 128.92, 128.61, 127.67, 127.06, 125.94, 125.24, 124.99, 117.92, 77.43, 77.11, 76.80, 52.41, 31.69, 29.80, 22.76, 14.23, 1.48, 1.12, 0.74. HRMS (ESI-TOF) m/z: $[M+H]^+$ calcd for $C_{46}H_{33}N_4O_4S$ 736.2144; Found 737.2263. $[\alpha]_D^{25}$ = 482.5 (*c* = 0.08, CH_2Cl_2).

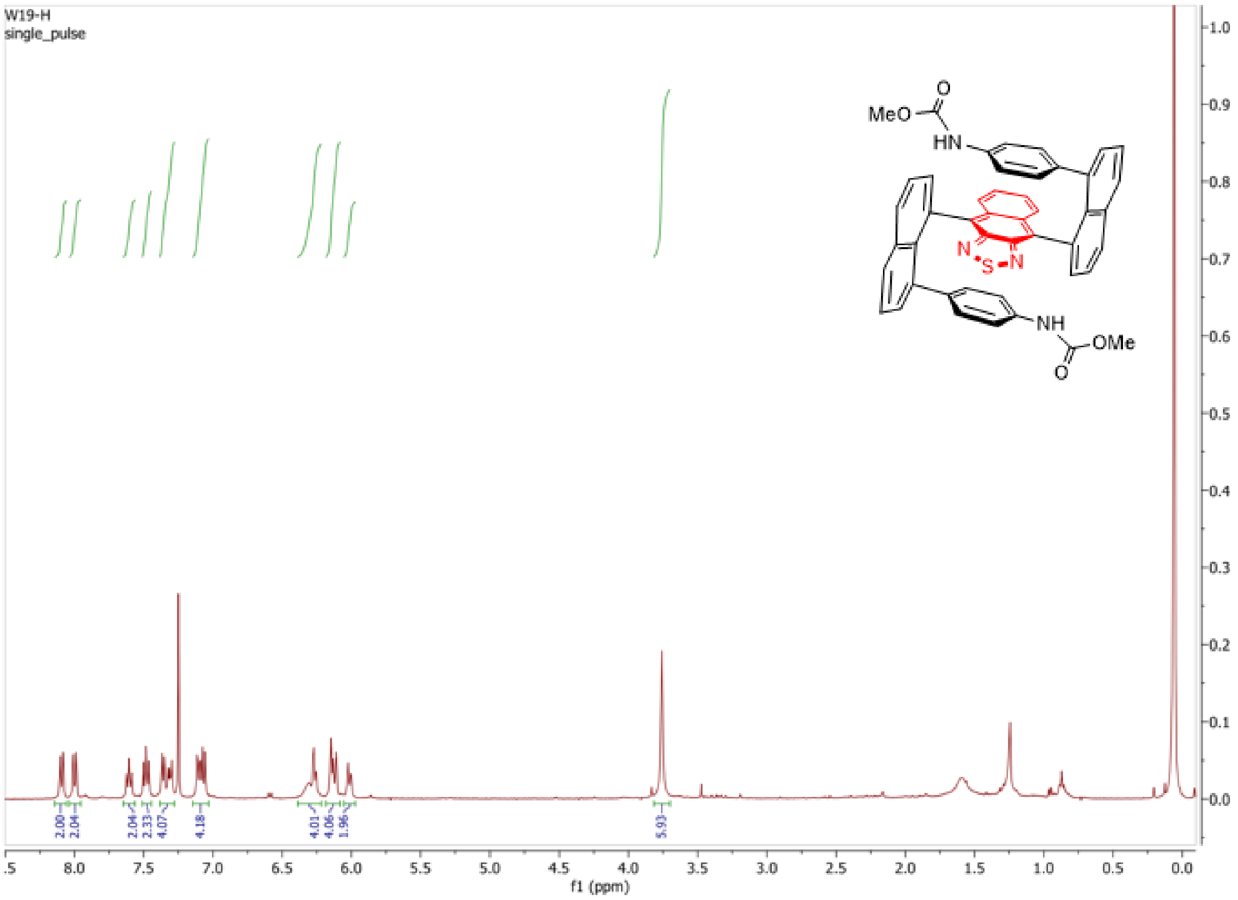

W19-H
single_pulse
MeO
O
HN
N
S
N
NH
OMe
O
2.00
2.04
2.04
2.33
4.07
4.18
4.01
4.06
1.96
5.93
f1 (ppm)

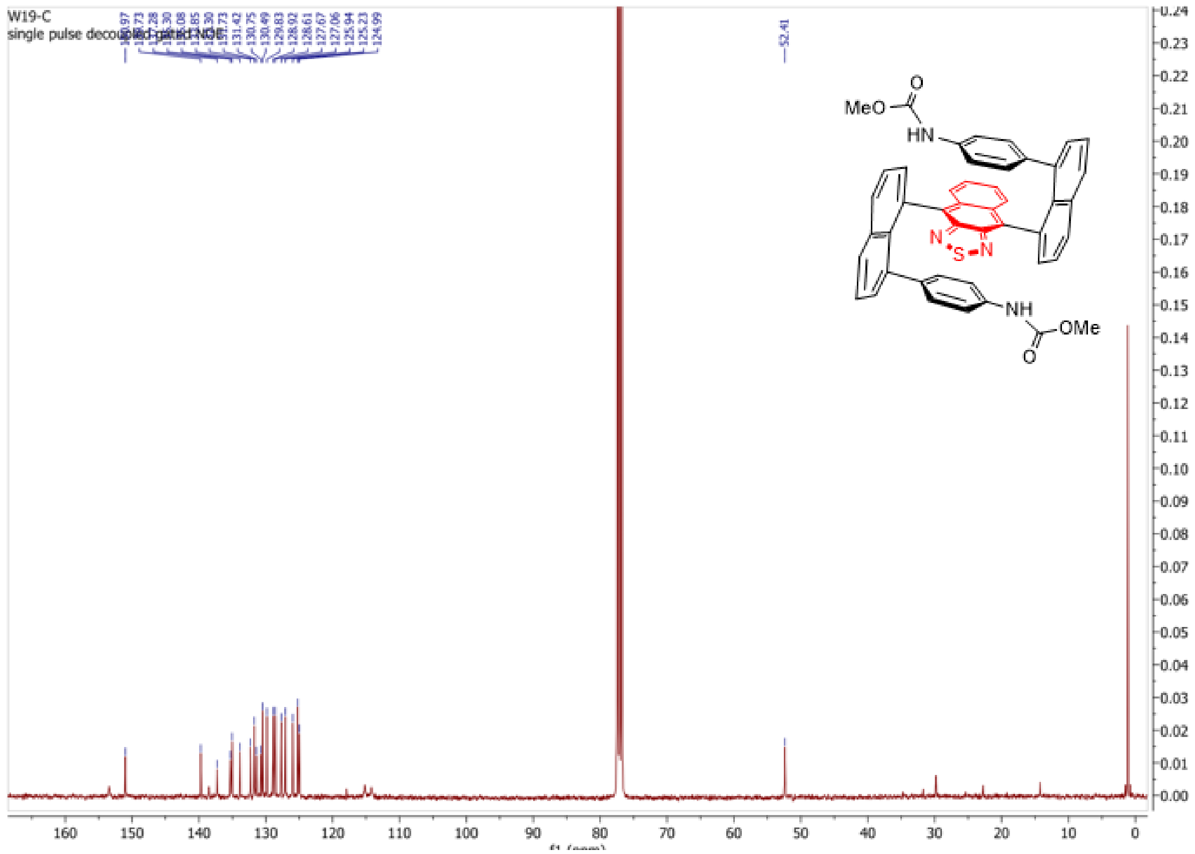

Crystal information

General Data Collection

Data were collected on a Rigaku XtaLAB Synergy-*i* Kappa diffractometer equipped with a PhotonJet-*i* X-ray source operated at 50 W (50kV, 1 mA) to generate Cu Kα radiation (λ = 1.54178 Å) and a HyPix-6000HE HPC detector. Crystals were transferred from the vial and placed on a glass slide in type NVH immersion oil by Cargille. A Zeiss Stemi 305 microscope was used to identify a suitable specimen for X-ray diffraction from a representative sample of the material. The crystal and a small amount of the oil were collected on a MiTeGen 50 micron MicroLoop and transferred to the instrument where it was placed under a cold nitrogen stream (Oxford 700 series) maintained at 100K throughout the duration of the experiment. The sample was optically centered with the aid of a video camera to insure that no translations were observed as the crystal was rotated through all positions.

A unit cell collection was then carried out. After it was determined that the unit cell was not present in the CCDC database a data collection strategy was calculated by $CrysAlis^{Pro}$[1]. The crystal was measured for size, morphology, and color. These values are reported in the accompanying Li21_05_tables file.

Refinement Details

After data collection, the unit cell was re-determined using a subset of the full data collection. Intensity data were corrected for Lorentz, polarization, and background effects using the

CrysAlis^{Pro}[1]. A numerical absorption correction was applied based on a Gaussian integration over a multifaceted crystal and followed by a semi-empirical correction for adsorption applied using the program *SCALE3 ABSPACK*[2]. The programs *SHELXT*[3] was used for the initial structure solution and *SHELXL*[4] was used for refinement of the structure. Both of these programs were utilized within the OLEX2 software[5]. The interstitial dichloromethane molecule was positionally disordered and modeled as two positions. To help maintain reasonable ADP values and bond lengths, SIMU, RIGU, and free variable DFIX restraints were applied to the disordered sites. A highly disordered hexane molecule was also present in the interstitial space and was treated with a solvent mask in the OLEX2 software[6]. Hydrogen atoms bound to carbon and nitrogen atoms were located in the difference Fourier map and were geometrically constrained using the appropriate AFIX commands. The Z' value for the final structure was 2.

References:

1. CrysAlis^{Pro} (2018) Oxford Diffraction Ltd.
2. SCALE3 ABSPACK (2005) Oxford Diffraction Ltd.
3. Sheldrick, G. M. (2015) *Acta Crystallogr.*, C**71**, 3-8.
4. Sheldrick, G. M. (2015) *Acta Crystallogr.*, A**71**, 3-8.
5. Dolomanov, O. V.; Bourhis, . L. J.; Gildea, R. J.; Howard, J. A. K.; Puschmann. H. (2009) *J. Appl. Cryst.* **42**, 339-341.
6. van der Sluis, P.; Spek, A.L. (1990) *Acta Crystallogr.,* **A46**, 194-201.

Li21_06

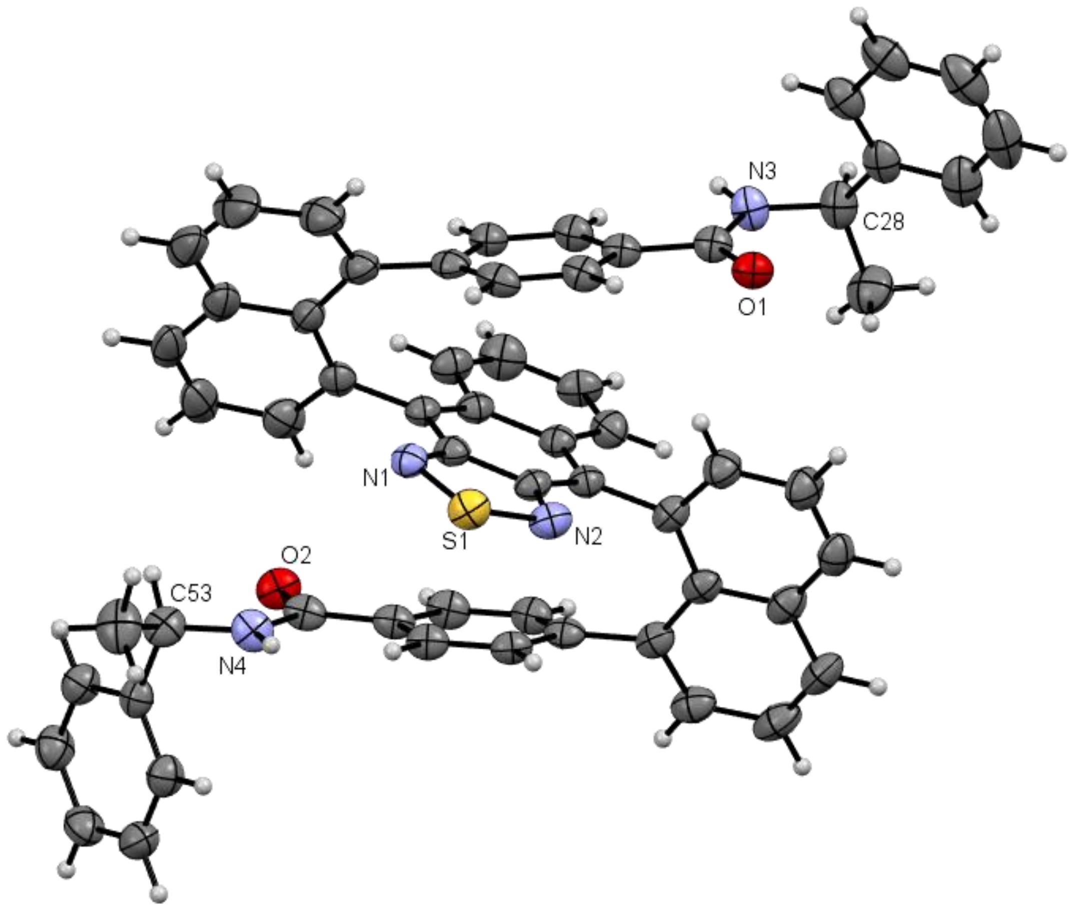

The thermal ellipsoids are represented at 50% probability. Carbon, hydrogen, nitrogen, oxygen and sulfur atoms are represented by gray, white, light blue, red and light orange ellipsoids, respectively. The interstitial dichloromethane molecule was omitted for clarity.

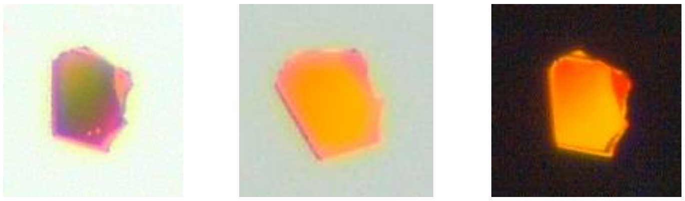

Crystal used for single crystal X-ray diffraction experiment.

Li21_06

Table 1 Crystal data and structure refinement for Li21_06.

Identification code	Li21_06
Empirical formula	$C_{60.5}H_{45}ClN_4O_2S$
Formula weight	927.51
Temperature/K	99.9(4)
Crystal system	triclinic
Space group	P1
a/Å	9.82240(10)
b/Å	14.3099(2)
c/Å	18.1202(3)
α/°	91.3630(10)
β/°	99.2700(10)
γ/°	99.6980(10)
Volume/$Å^3$	2474.35(6)
Z	2
$\rho_{calc}g/cm^3$	1.245
μ/mm^{-1}	1.453
F(000)	970.0
Crystal size/mm^3	0.151 × 0.122 × 0.015

Radiation	Cu Kα (λ = 1.54184)
2Θ range for data collection/°	4.948 to 134.16
Index ranges	-11 ≤ h ≤ 11, -17 ≤ k ≤ 17, -21 ≤ l ≤ 21
Reflections collected	99732
Independent reflections	17143 [R_{int} = 0.0557, R_{sigma} = 0.0369]
Data/restraints/parameters	17143/81/1267
Goodness-of-fit on F^2	1.050
Final R indexes [I>=2σ (I)]	R_1 = 0.0495, wR_2 = 0.1293
Final R indexes [all data]	R_1 = 0.0569, wR_2 = 0.1366
Largest diff. peak/hole / e $Å^{-3}$	0.34/-0.33
Flack parameter	0.026(13)

Table 2 Fractional Atomic Coordinates (×10^4) and Equivalent Isotropic Displacement Parameters ($Å^2$×10^3) for Li21_06. U_{eq} is defined as 1/3 of of the trace of the orthogonalised U_{IJ} tensor.

Atom	*x*	*y*	*z*	U(eq)
S1	11083.0(11)	3668.0(8)	2965.2(7)	43.3(3)

Table 2 Fractional Atomic Coordinates (×10⁴) and Equivalent Isotropic Displacement Parameters (Å²×10³) for Li21_06. U_{eq} is defined as 1/3 of of the trace of the orthogonalised U_{IJ} tensor.

Atom	*x*	*y*	*z*	U(eq)
O1	8525(3)	1479(2)	-14.5(16)	38.5(7)
O2	5548(3)	5470(2)	4474.3(18)	41.3(7)
N1	10211(4)	4512(2)	2787.3(19)	35.3(8)
N2	9866(4)	2738(3)	2806(2)	39.9(8)
N3	6364(4)	1751(3)	-473(2)	39.9(8)
N4	7851(4)	5591(3)	4863(2)	39.6(8)
C1	7709(4)	4561(3)	2385(2)	31.4(8)
C2	8858(4)	4085(3)	2590(2)	32.6(8)
C3	8660(4)	3069(3)	2595(2)	32.9(9)
C4	7312(4)	2500(3)	2412(2)	32.5(9)
C5	6182(4)	2970(3)	2201(2)	34.2(9)
C6	6381(4)	4002(3)	2204(2)	33.2(9)
C7	5173(5)	4423(3)	1963(3)	42.4(10)
C8	3894(5)	3900(4)	1745(3)	52.6(12)
C9	3734(5)	2936(3)	1738(3)	42.3(10)

Table 2 Fractional Atomic Coordinates (×10⁴) and Equivalent Isotropic Displacement Parameters (Å²×10³) for Li21_06. U_{eq} is defined as 1/3 of of the trace of the orthogonalised U_{IJ} tensor.

Atom	*x*	*y*	*z*	U(eq)
C10	4801(5)	2455(3)	1953(3)	42.2(10)
C11	7947(5)	5616(3)	2409(2)	36.9(9)
C12	7403(6)	6057(4)	2952(3)	48.9(11)
C13	7708(7)	7051(4)	3095(3)	58.3(14)
C14	8607(6)	7593(3)	2714(3)	56.0(13)
C15	9153(6)	7183(3)	2136(3)	49.7(12)
C16	8797(5)	6182(3)	1954(2)	39.0(9)
C17	9352(5)	5800(3)	1341(3)	43.5(10)
C18	10306(6)	6401(4)	1015(3)	59.2(14)
C19	10692(7)	7371(4)	1226(4)	73.0(19)
C20	10099(7)	7764(4)	1758(3)	64.8(16)
C21	8880(5)	4818(3)	1002(2)	36.4(9)
C22	9846(5)	4226(3)	940(2)	40.3(10)
C23	9420(5)	3331(3)	596(2)	38.2(10)
C24	8019(4)	2996(3)	300(2)	33.4(9)

Table 2 Fractional Atomic Coordinates (×10⁴) and Equivalent Isotropic Displacement Parameters (Å²×10³) for Li21_06. U_{eq} is defined as 1/3 of of the trace of the orthogonalised U_{IJ} tensor.

Atom	*x*	*y*	*z*	U(eq)
C25	7045(5)	3591(3)	350(2)	37.3(9)
C26	7479(5)	4495(3)	694(2)	35.9(9)
C27	7647(4)	2015(3)	-73(2)	34.6(9)
C28	5795(5)	791(3)	-807(3)	45.6(11)
C29	6684(5)	482(3)	-1353(3)	41.7(10)
C30	7393(5)	1131(4)	-1776(3)	45.9(11)
C31	8186(5)	833(4)	-2288(3)	55.8(13)
C32	8243(6)	-116(5)	-2386(3)	63.0(15)
C33	7518(6)	-757(4)	-1996(4)	69.4(18)
C34	6744(6)	-473(4)	-1473(3)	58.3(14)
C35	5548(7)	120(4)	-187(4)	63.5(15)
C36	7180(4)	1445(3)	2390(3)	35.7(9)
C37	7689(5)	1055(3)	1810(3)	44.3(11)
C38	7591(6)	82(3)	1687(3)	48.0(11)
C39	6982(5)	-520(3)	2152(3)	45.3(11)

Table 2 Fractional Atomic Coordinates (×10[4]) and Equivalent Isotropic Displacement Parameters (Å^2×10^3) for Li21_06. U_{eq} is defined as 1/3 of of the trace of the orthogonalised U_{IJ} tensor.

Atom	*x*	*y*	*z*	U(eq)
C40	6467(5)	-168(3)	2770(3)	42.4(10)
C41	6571(4)	830(3)	2914(2)	37.2(9)
C42	6039(4)	1142(3)	3554(2)	36.9(9)
C43	5392(5)	475(3)	3985(3)	44.9(10)
C44	5299(5)	-507(4)	3833(3)	51.1(12)
C45	5839(5)	-818(3)	3251(3)	48.9(12)
C46	6194(4)	2160(3)	3812(2)	35.1(9)
C47	5024(4)	2591(3)	3814(2)	38.9(10)
C48	5173(4)	3527(3)	4057(3)	38.0(9)
C49	6503(4)	4075(3)	4319(2)	34.8(9)
C50	7673(4)	3627(3)	4337(2)	36.3(9)
C51	7507(4)	2685(3)	4093(2)	33.9(9)
C52	6604(4)	5087(3)	4555(2)	35.2(9)
C53	7958(5)	6570(3)	5140(3)	42.1(10)
C54	7345(4)	6608(3)	5862(3)	35.4(9)

Table 2 Fractional Atomic Coordinates (×10⁴) and Equivalent Isotropic Displacement Parameters (Å²×10³) for Li21_06. U_{eq} is defined as 1/3 of of the trace of the orthogonalised U_{IJ} tensor.

Atom	*x*	*y*	*z*	U(eq)
C55	6512(5)	7272(3)	5971(3)	43.1(10)
C56	5964(6)	7316(3)	6620(3)	49.9(12)
C57	6239(6)	6697(3)	7175(3)	49.4(12)
C58	7076(6)	6037(3)	7078(3)	49.4(11)
C59	7634(5)	5992(3)	6420(3)	43.9(10)
C60	9479(6)	7065(4)	5260(4)	60.1(14)
S2	-1096.3(12)	3129.5(9)	6360.4(8)	50.9(3)
O3	602(3)	5168(2)	4688.2(18)	40.3(7)
O4	3588(3)	1926(2)	9777.5(17)	39.7(7)
N5	51(4)	2447(3)	6580(2)	43.2(9)
N6	-295(4)	4134(3)	6738(2)	41.5(9)
N7	2737(4)	5678(2)	4382(2)	36.5(8)
N8	1311(3)	1243(2)	9745(2)	33.2(7)
C61	2462(4)	2733(3)	7287(2)	32.6(9)
C62	1187(4)	3018(3)	6992(2)	33.2(9)

Table 2 Fractional Atomic Coordinates (×10^4) and Equivalent Isotropic Displacement Parameters (Å^2×10^3) for Li21_06. U_{eq} is defined as 1/3 of of the trace of the orthogonalised U_{IJ} tensor.

Atom	*x*	*y*	*z*	U(eq)
C63	982(4)	3980(3)	7080(2)	33.9(9)
C64	2030(4)	4672(3)	7503(2)	32.7(9)
C65	3282(4)	4394(3)	7804(2)	32.1(8)
C66	3502(4)	3417(3)	7699(2)	30.6(8)
C67	4814(4)	3198(3)	8046(2)	34.9(9)
C68	5800(5)	3885(4)	8457(2)	46.0(11)
C69	5594(5)	4772(4)	8564(3)	48.9(12)
C70	4391(5)	5040(3)	8258(2)	36.4(9)
C71	2682(4)	1732(3)	7199(2)	33.2(9)
C72	2086(5)	1080(3)	7648(3)	41.9(10)
C73	2444(6)	169(3)	7700(3)	46.6(11)
C74	3457(5)	-57(3)	7336(3)	45.3(11)
C75	4096(5)	588(3)	6859(2)	38.8(10)
C76	3656(4)	1480(3)	6747(2)	34.1(9)
C77	4234(4)	2080(3)	6214(2)	33.9(9)

Table 2 Fractional Atomic Coordinates (×10⁴) and Equivalent Isotropic Displacement Parameters (Å²×10³) for Li21_06. U_{eq} is defined as 1/3 of of the trace of the orthogonalised U_{IJ} tensor.

Atom	*x*	*y*	*z*	U(eq)
C78	5305(5)	1835(3)	5891(3)	40.4(10)
C79	5821(5)	988(3)	6052(3)	43.7(11)
C80	5196(5)	371(3)	6505(3)	44.5(11)
C81	3611(4)	2908(3)	5909(2)	31.0(8)
C82	2285(4)	2727(3)	5468(2)	37.6(9)
C83	1724(4)	3448(3)	5114(2)	36.5(9)
C84	2476(4)	4374(3)	5186(2)	31.3(9)
C85	3791(4)	4556(3)	5630(2)	31.3(8)
C86	4355(4)	3834(3)	5997(2)	34.5(9)
C87	1846(4)	5116(3)	4740(2)	33.3(9)
C88	2302(4)	6395(3)	3881(2)	36.1(9)
C89	2435(4)	7360(3)	4277(2)	35.5(9)
C90	1850(5)	8067(3)	3892(3)	45.3(11)
C91	1927(6)	8958(4)	4233(3)	53.6(13)
C92	2593(6)	9156(4)	4964(3)	51.0(12)

Table 2 Fractional Atomic Coordinates (×10⁴) and Equivalent Isotropic Displacement Parameters (Å²×10³) for Li21_06. U_{eq} is defined as 1/3 of of the trace of the orthogonalised U_{IJ} tensor.

Atom	*x*	*y*	*z*	U(eq)
C93	3174(6)	8476(3)	5346(3)	53.5(13)
C94	3102(5)	7571(3)	4998(3)	45.1(11)
C95	3155(6)	6454(4)	3238(3)	50.7(12)
C96	1776(4)	5669(3)	7565(2)	35.9(9)
C97	2275(6)	6284(3)	7063(3)	47.4(11)
C98	1955(6)	7207(3)	7012(3)	56.5(13)
C99	1133(6)	7499(3)	7471(3)	53.2(13)
C100	599(5)	6899(3)	8005(3)	43.2(10)
C101	943(5)	5974(3)	8079(3)	36.7(9)
C102	429(4)	5416(3)	8650(2)	36.5(9)
C103	-468(5)	5747(3)	9072(3)	44.9(11)
C104	-829(6)	6651(4)	8981(3)	52.6(12)
C105	-288(6)	7224(3)	8475(3)	51.0(12)
C106	908(4)	4511(3)	8889(2)	32.5(9)
C107	96(4)	3627(3)	8662(2)	33.8(9)

Table 2 Fractional Atomic Coordinates (×10^4) and Equivalent Isotropic Displacement Parameters (Å^2×10^3) for Li21_06. U$_{eq}$ is defined as 1/3 of of the trace of the orthogonalised U$_{IJ}$ tensor.

Atom	*x*	*y*	*z*	U(eq)
C108	538(4)	2808(3)	8907(2)	31.6(8)
C109	1820(4)	2848(3)	9391(2)	30.9(8)
C110	2630(4)	3731(3)	9623(2)	33.4(9)
C111	2158(4)	4554(3)	9376(2)	34.5(9)
C112	2330(4)	1973(3)	9658(2)	32.0(8)
C113	1603(5)	327(3)	10005(3)	37.6(9)
C114	1852(4)	304(3)	10846(3)	39.3(10)
C115	1107(5)	762(3)	11286(3)	45.3(11)
C116	1310(7)	714(4)	12057(3)	61.9(15)
C117	2270(6)	209(4)	12399(3)	64.8(16)
C118	3014(6)	-264(5)	11976(4)	69.3(18)
C119	2818(5)	-220(3)	11204(3)	51.3(12)
C120	372(6)	-437(3)	9662(3)	51.2(12)
Cl1	9451(3)	10334(2)	5518.9(17)	97.1(11)
Cl2	9893(2)	10833.5(15)	4023.6(15)	68.0(7)

Table 2 Fractional Atomic Coordinates (×10⁴) and Equivalent Isotropic Displacement Parameters (Å²×10³) for Li21_06. U_{eq} is defined as 1/3 of of the trace of the orthogonalised U_{IJ} tensor.

Atom	*x*	*y*	*z*	U(eq)
C121	9065(11)	9995(8)	4542(6)	72(2)
Cl4	8501(9)	9373(5)	5702(6)	127(3)
Cl3	9469(7)	10649(5)	4622(6)	105(3)
C122	8990(40)	9544(19)	4870(17)	123(6)

Table 3 Anisotropic Displacement Parameters (Å²×10³) for Li21_06. The Anisotropic displacement factor exponent takes the form: $-2\pi^2[h^2a^{*2}U_{11}+2hka^*b^*U_{12}+\ldots]$.

Atom	U_{11}	U_{22}	U_{33}	U_{23}	U_{13}	U_{12}
S1	31.9(6)	48.6(6)	49.4(6)	5.9(5)	5.0(5)	8.2(4)
O1	30.2(15)	47.6(16)	39.3(16)	5.2(13)	6.3(13)	10.4(13)
O2	23.4(15)	52.3(17)	51.9(18)	8.2(14)	10.6(14)	11.8(13)
N1	32.4(19)	37.8(18)	34.7(18)	5.0(14)	7.8(15)	1.0(15)
N2	34(2)	47(2)	42(2)	10.2(16)	10.4(17)	10.4(16)
N3	23.7(18)	44(2)	51(2)	-6.1(17)	2.4(16)	7.1(15)

Table 3 Anisotropic Displacement Parameters (Å^2×10^3) for Li21_06. The Anisotropic displacement factor exponent takes the form: $-2\pi^2[h^2a^{*2}U_{11}+2hka^*b^*U_{12}+\ldots]$.

Atom	U_{11}	U_{22}	U_{33}	U_{23}	U_{13}	U_{12}
N4	22.0(18)	51(2)	48(2)	-0.3(17)	8.3(16)	10.3(15)
C1	32(2)	34(2)	29(2)	4.8(16)	7.7(17)	6.0(16)
C2	33(2)	37(2)	27.5(19)	5.1(16)	6.9(17)	3.2(17)
C3	33(2)	36(2)	33(2)	7.3(16)	10.4(17)	8.5(17)
C4	30(2)	36(2)	32(2)	5.0(16)	7.9(17)	6.3(16)
C5	33(2)	38(2)	32(2)	5.2(16)	8.7(18)	4.1(17)
C6	33(2)	38(2)	31(2)	5.4(16)	9.5(18)	8.5(17)
C7	38(3)	48(2)	45(2)	8.4(19)	7(2)	16(2)
C8	33(3)	63(3)	61(3)	3(2)	6(2)	9(2)
C9	27(2)	57(3)	44(2)	11(2)	3.6(19)	11.8(19)
C10	39(3)	43(2)	43(2)	2.1(19)	9(2)	0.2(19)
C11	35(2)	38(2)	36(2)	2.3(17)	-1.6(18)	9.9(18)
C12	51(3)	52(3)	48(3)	5(2)	5(2)	24(2)
C13	73(4)	48(3)	55(3)	-6(2)	-10(3)	34(3)
C14	68(4)	34(2)	57(3)	-1(2)	-21(3)	15(2)

Table 3 Anisotropic Displacement Parameters (Å^2×10^3) for Li21_06. The Anisotropic displacement factor exponent takes the form: $-2\pi^2[h^2a^{*2}U_{11}+2hka^*b^*U_{12}+...]$.

Atom	U_{11}	U_{22}	U_{33}	U_{23}	U_{13}	U_{12}
C15	55(3)	37(2)	49(3)	5(2)	-13(2)	5(2)
C16	40(2)	31(2)	42(2)	5.7(17)	-3(2)	2.8(18)
C17	44(3)	43(2)	38(2)	10.1(19)	2(2)	-5.0(19)
C18	60(3)	62(3)	48(3)	11(2)	7(3)	-12(3)
C19	76(4)	59(3)	64(4)	17(3)	-3(3)	-31(3)
C20	74(4)	41(3)	63(3)	12(2)	-19(3)	-9(3)
C21	35(2)	44(2)	28(2)	10.5(17)	8.1(18)	-1.6(18)
C22	26(2)	61(3)	31(2)	3.7(19)	3.9(17)	-0.7(19)
C23	29(2)	50(2)	36(2)	2.5(18)	7.9(18)	4.1(18)
C24	26(2)	43(2)	33(2)	4.9(17)	8.1(17)	7.6(17)
C25	25(2)	47(2)	37(2)	3.1(18)	3.3(18)	2.6(18)
C26	35(2)	40(2)	32(2)	4.8(17)	6.2(18)	6.7(17)
C27	25(2)	42(2)	38(2)	3.4(17)	9.5(18)	5.0(17)
C28	30(2)	43(2)	62(3)	-6(2)	4(2)	4.9(18)
C29	26(2)	46(2)	48(3)	-6(2)	-8.5(19)	8.5(18)

Table 3 Anisotropic Displacement Parameters (Å^2×10^3) for Li21_06. The Anisotropic displacement factor exponent takes the form: $-2\pi^2[h^2a^{*2}U_{11}+2hka^*b^*U_{12}+...]$.

Atom	U_{11}	U_{22}	U_{33}	U_{23}	U_{13}	U_{12}
C30	32(2)	56(3)	45(3)	-7(2)	-3(2)	5(2)
C31	34(3)	80(4)	48(3)	-9(3)	-4(2)	5(2)
C32	43(3)	87(4)	57(3)	-23(3)	-5(3)	22(3)
C33	54(3)	61(3)	86(4)	-27(3)	-24(3)	29(3)
C34	48(3)	51(3)	71(4)	-9(3)	-11(3)	16(2)
C35	56(3)	59(3)	73(4)	9(3)	17(3)	-5(3)
C36	32(2)	33(2)	44(2)	8.0(17)	7.5(19)	6.2(17)
C37	47(3)	41(2)	46(3)	5.5(19)	13(2)	6(2)
C38	53(3)	41(2)	55(3)	2(2)	13(2)	17(2)
C39	43(3)	36(2)	57(3)	3(2)	1(2)	15.0(19)
C40	34(2)	39(2)	54(3)	11.0(19)	3(2)	7.4(18)
C41	29(2)	40(2)	41(2)	8.8(18)	1.3(18)	6.2(17)
C42	28(2)	40(2)	42(2)	8.7(18)	4.0(18)	2.5(17)
C43	35(2)	52(3)	46(3)	13(2)	8(2)	3(2)
C44	38(3)	50(3)	63(3)	26(2)	6(2)	0(2)

Table 3 Anisotropic Displacement Parameters ($Å^2×10^3$) for Li21_06. The Anisotropic displacement factor exponent takes the form: $-2\pi^2[h^2a^{*2}U_{11}+2hka^*b^*U_{12}+\ldots]$.

Atom	U_{11}	U_{22}	U_{33}	U_{23}	U_{13}	U_{12}
C45	42(3)	38(2)	66(3)	20(2)	5(2)	5(2)
C46	30(2)	46(2)	30(2)	7.7(17)	10.2(17)	4.4(17)
C47	25(2)	50(2)	39(2)	4.2(19)	5.9(18)	0.1(18)
C48	21(2)	53(3)	42(2)	4.0(19)	7.9(18)	10.3(18)
C49	24(2)	49(2)	33(2)	5.7(17)	8.3(17)	8.6(17)
C50	23(2)	50(2)	38(2)	6.6(18)	8.9(18)	7.8(17)
C51	25(2)	45(2)	34(2)	9.3(17)	7.3(17)	11.3(17)
C52	19(2)	53(2)	35(2)	4.4(18)	9.2(17)	7.5(17)
C53	32(2)	47(2)	50(3)	6(2)	17(2)	6.6(18)
C54	25(2)	34(2)	45(2)	0.0(17)	4.3(18)	1.1(16)
C55	39(2)	42(2)	50(3)	4(2)	9(2)	10.0(19)
C56	51(3)	46(3)	56(3)	-1(2)	18(2)	11(2)
C57	57(3)	50(3)	42(3)	-3(2)	17(2)	4(2)
C58	57(3)	44(2)	45(3)	9(2)	6(2)	6(2)
C59	41(3)	42(2)	51(3)	1(2)	8(2)	11.2(19)

Table 3 Anisotropic Displacement Parameters (Å²×10³) for Li21_06. The Anisotropic displacement factor exponent takes the form: $-2\pi^2[h^2a^{*2}U_{11}+2hka^*b^*U_{12}+\ldots]$.

Atom	U_{11}	U_{22}	U_{33}	U_{23}	U_{13}	U_{12}
C60	41(3)	60(3)	81(4)	-4(3)	27(3)	0(2)
S2	34.6(6)	49.7(6)	63.4(8)	6.0(5)	-4.9(5)	5.7(5)
O3	22.5(15)	52.0(17)	50.6(18)	13.2(14)	11.8(13)	10.9(12)
O4	26.7(16)	45.4(16)	49.6(18)	8.1(13)	9.2(13)	10.2(12)
N5	36(2)	37.7(19)	51(2)	7.6(16)	-0.2(17)	0.2(16)
N6	32(2)	44(2)	49(2)	11.5(17)	3.7(17)	9.9(16)
N7	20.1(16)	42.6(19)	48(2)	14.9(16)	8.0(15)	5.8(14)
N8	24.1(17)	33.4(17)	44.3(19)	10.6(14)	7.4(15)	8.3(13)
C61	29(2)	36(2)	31(2)	5.7(16)	5.1(17)	3.6(16)
C62	29(2)	34(2)	36(2)	4.9(16)	4.8(17)	3.2(16)
C63	31(2)	36(2)	36(2)	8.3(17)	8.0(18)	6.5(17)
C64	31(2)	33(2)	35(2)	8.0(16)	8.0(18)	3.2(16)
C65	29(2)	32.6(19)	34(2)	5.3(16)	7.8(17)	2.3(16)
C66	30(2)	30.6(19)	31(2)	5.3(15)	6.5(17)	1.5(15)
C67	33(2)	37(2)	36(2)	4.7(17)	6.4(18)	8.5(17)

Table 3 Anisotropic Displacement Parameters (Å^2×10^3) for Li21_06. The Anisotropic displacement factor exponent takes the form: -2π^2[h^{2}a*^{2}U$_{11}$+2hka*b*U$_{12}$+…].

Atom	U_{11}	U_{22}	U_{33}	U_{23}	U_{13}	U_{12}
C68	25(2)	73(3)	32(2)	7(2)	-3.1(18)	-8(2)
C69	28(2)	75(3)	38(2)	10(2)	0(2)	-3(2)
C70	36(2)	32(2)	38(2)	2.8(17)	6.4(19)	-2.4(17)
C71	27(2)	34(2)	36(2)	2.3(16)	-1.7(17)	4.7(16)
C72	41(3)	37(2)	46(2)	6.2(18)	4(2)	4.3(19)
C73	53(3)	32(2)	49(3)	4.4(19)	3(2)	-2.5(19)
C74	52(3)	26(2)	52(3)	2.6(18)	-6(2)	6.0(19)
C75	40(2)	33(2)	39(2)	-5.5(17)	-5.9(19)	8.7(18)
C76	30(2)	32(2)	36(2)	0.5(16)	-3.6(17)	3.6(16)
C77	27(2)	37(2)	35(2)	-1.9(16)	-3.2(17)	4.1(16)
C78	32(2)	46(2)	42(2)	-2.7(19)	1.6(19)	6.7(18)
C79	32(2)	54(3)	44(3)	-10(2)	-2(2)	15(2)
C80	44(3)	34(2)	50(3)	-9.9(19)	-11(2)	13.2(19)
C81	24(2)	38(2)	32(2)	4.4(16)	4.8(17)	8.0(16)
C82	28(2)	39(2)	42(2)	3.6(18)	-0.1(19)	2.9(17)

Table 3 Anisotropic Displacement Parameters (Å^2×10^3) for Li21_06. The Anisotropic displacement factor exponent takes the form: $-2\pi^2[h^2a^{*2}U_{11}+2hka^*b^*U_{12}+...]$.

Atom	U_{11}	U_{22}	U_{33}	U_{23}	U_{13}	U_{12}
C83	19(2)	45(2)	44(2)	8.7(18)	0.9(18)	3.0(16)
C84	23(2)	38(2)	36(2)	6.2(16)	12.3(17)	5.7(16)
C85	23(2)	35(2)	38(2)	2.4(16)	9.2(17)	4.7(16)
C86	22(2)	43(2)	37(2)	0.2(17)	4.3(17)	1.4(16)
C87	21(2)	42(2)	38(2)	6.1(17)	6.4(17)	7.2(16)
C88	25(2)	42(2)	42(2)	10.2(18)	4.6(18)	5.9(17)
C89	20.8(19)	45(2)	40(2)	12.2(18)	6.4(17)	1.3(16)
C90	40(3)	51(3)	47(3)	10(2)	2(2)	16(2)
C91	47(3)	52(3)	66(3)	16(2)	12(3)	19(2)
C92	49(3)	45(3)	60(3)	4(2)	21(3)	0(2)
C93	61(3)	43(3)	46(3)	9(2)	0(2)	-13(2)
C94	41(3)	41(2)	47(3)	14.9(19)	1(2)	-5.7(19)
C95	53(3)	54(3)	48(3)	11(2)	17(2)	9(2)
C96	33(2)	31(2)	43(2)	7.6(17)	4.7(19)	3.1(17)
C97	52(3)	37(2)	55(3)	10(2)	15(2)	5(2)

Table 3 Anisotropic Displacement Parameters (Å²×10³) for Li21_06. The Anisotropic displacement factor exponent takes the form: $-2\pi^2[h^2a^{*2}U_{11}+2hka^*b^*U_{12}+...]$.

Atom	U_{11}	U_{22}	U_{33}	U_{23}	U_{13}	U_{12}
C98	66(3)	39(3)	65(3)	19(2)	14(3)	4(2)
C99	64(3)	29(2)	63(3)	10(2)	2(3)	7(2)
C100	43(3)	31(2)	53(3)	0.2(19)	-4(2)	11.1(18)
C101	33(2)	30(2)	44(2)	2.9(17)	-0.6(19)	4.0(17)
C102	31(2)	39(2)	40(2)	3.3(17)	2.1(18)	10.6(17)
C103	41(3)	52(3)	45(3)	3(2)	6(2)	18(2)
C104	51(3)	56(3)	53(3)	-12(2)	3(2)	23(2)
C105	52(3)	40(2)	62(3)	-1(2)	0(2)	21(2)
C106	31(2)	34(2)	36(2)	6.5(16)	11.5(18)	9.9(16)
C107	25(2)	43(2)	32(2)	5.7(17)	2.2(17)	6.6(17)
C108	28(2)	31.4(19)	36(2)	3.3(16)	7.6(17)	2.2(15)
C109	26(2)	37(2)	33(2)	6.4(16)	11.7(17)	6.1(16)
C110	25(2)	40(2)	34(2)	8.8(17)	3.8(17)	4.3(17)
C111	30(2)	34(2)	39(2)	3.5(17)	6.4(18)	4.5(16)
C112	26(2)	41(2)	32(2)	4.9(16)	7.1(17)	13.1(17)

Table 3 Anisotropic Displacement Parameters (Å^2×10^3) for Li21_06. The Anisotropic displacement factor exponent takes the form: $-2\pi^2[h^2a^{*2}U_{11}+2hka^*b^*U_{12}+...]$.

Atom	U_{11}	U_{22}	U_{33}	U_{23}	U_{13}	U_{12}
C113	33(2)	30(2)	50(3)	6.0(18)	6(2)	8.9(17)
C114	30(2)	32(2)	52(3)	12.2(18)	2(2)	-1.2(17)
C115	45(3)	46(2)	44(3)	10(2)	7(2)	7(2)
C116	71(4)	61(3)	43(3)	6(2)	-3(3)	-7(3)
C117	58(4)	69(4)	53(3)	20(3)	-5(3)	-18(3)
C118	42(3)	74(4)	79(4)	43(3)	-14(3)	-10(3)
C119	32(2)	50(3)	68(3)	29(2)	2(2)	1(2)
C120	56(3)	39(2)	55(3)	2(2)	-4(2)	9(2)
Cl1	79.2(18)	124(2)	87.2(18)	-21.9(15)	36.7(14)	-3.6(15)
Cl2	57.7(13)	62.0(12)	89.5(17)	10.1(11)	10.1(12)	27.0(10)
C121	53(5)	59(5)	104(7)	0(5)	20(4)	2(4)
Cl4	118(6)	86(4)	183(7)	-32(4)	56(5)	13(4)
Cl3	69(4)	64(4)	166(7)	10(4)	-27(4)	11(3)
C122	92(10)	107(11)	163(11)	-20(9)	12(9)	14(8)

Table 4 Bond Lengths for Li21_06.

Atom	Atom	Length/Å	Atom	Atom	Length/Å
S1	N1	1.605(4)	O3	C87	1.225(5)
S1	N2	1.618(4)	O4	C112	1.233(5)
O1	C27	1.241(5)	N5	C62	1.366(6)
O2	C52	1.242(5)	N6	C63	1.362(6)
N1	C2	1.354(5)	N7	C87	1.344(5)
N2	C3	1.350(6)	N7	C88	1.458(5)
N3	C27	1.337(6)	N8	C112	1.352(5)
N3	C28	1.469(6)	N8	C113	1.461(5)
N4	C52	1.340(6)	C61	C62	1.410(6)
N4	C53	1.458(6)	C61	C66	1.394(6)
C1	C2	1.419(6)	C61	C71	1.492(6)
C1	C6	1.395(6)	C62	C63	1.434(6)
C1	C11	1.487(6)	C63	C64	1.412(6)
C2	C3	1.435(6)	C64	C65	1.388(6)
C3	C4	1.417(6)	C64	C96	1.493(6)
C4	C5	1.400(6)	C65	C66	1.463(5)

Table 4 Bond Lengths for Li21_06.

Atom	Atom	Length/Å	Atom	Atom	Length/Å
C4	C36	1.492(6)	C65	C70	1.433(6)
C5	C6	1.457(6)	C66	C67	1.429(6)
C5	C10	1.427(6)	C67	C68	1.368(6)
C6	C7	1.430(6)	C68	C69	1.332(8)
C7	C8	1.343(7)	C69	C70	1.342(7)
C8	C9	1.361(7)	C71	C72	1.371(6)
C9	C10	1.360(7)	C71	C76	1.438(6)
C11	C12	1.380(6)	C72	C73	1.408(7)
C11	C16	1.435(6)	C73	C74	1.356(7)
C12	C13	1.413(7)	C74	C75	1.418(7)
C13	C14	1.359(9)	C75	C76	1.424(6)
C14	C15	1.409(8)	C75	C80	1.414(7)
C15	C16	1.434(6)	C76	C77	1.431(6)
C15	C20	1.414(8)	C77	C78	1.373(6)
C16	C17	1.448(7)	C77	C81	1.499(6)
C17	C18	1.377(6)	C78	C79	1.410(7)
C17	C21	1.488(6)	C79	C80	1.359(7)

Table 4 Bond Lengths for Li21_06.

Atom	Atom	Length/Å	Atom	Atom	Length/Å
C18	C19	1.403(8)	C81	C82	1.394(6)
C19	C20	1.360(10)	C81	C86	1.394(6)
C21	C22	1.390(6)	C82	C83	1.375(6)
C21	C26	1.394(6)	C83	C84	1.396(6)
C22	C23	1.379(6)	C84	C85	1.387(6)
C23	C24	1.393(6)	C84	C87	1.504(6)
C24	C25	1.395(6)	C85	C86	1.387(6)
C24	C27	1.504(6)	C88	C89	1.517(6)
C25	C26	1.391(6)	C88	C95	1.537(6)
C28	C29	1.526(6)	C89	C90	1.393(6)
C28	C35	1.517(8)	C89	C94	1.366(7)
C29	C30	1.387(7)	C90	C91	1.388(7)
C29	C34	1.391(7)	C91	C92	1.381(8)
C30	C31	1.405(7)	C92	C93	1.357(8)
C31	C32	1.377(9)	C93	C94	1.413(7)
C32	C33	1.349(9)	C96	C97	1.371(6)
C33	C34	1.401(9)	C96	C101	1.440(6)

Table 4 Bond Lengths for Li21_06.

Atom	Atom	Length/Å	Atom	Atom	Length/Å
C36	C37	1.379(6)	C97	C98	1.410(7)
C36	C41	1.442(6)	C98	C99	1.354(8)
C37	C38	1.390(7)	C99	C100	1.414(7)
C38	C39	1.354(7)	C100	C101	1.425(6)
C39	C40	1.415(7)	C100	C105	1.434(7)
C40	C41	1.430(6)	C101	C102	1.425(6)
C40	C45	1.423(6)	C102	C103	1.387(6)
C41	C42	1.436(6)	C102	C106	1.500(6)
C42	C43	1.385(6)	C103	C104	1.405(7)
C42	C46	1.495(6)	C104	C105	1.358(8)
C43	C44	1.411(7)	C106	C107	1.391(6)
C44	C45	1.353(7)	C106	C111	1.384(6)
C46	C47	1.393(6)	C107	C108	1.376(6)
C46	C51	1.389(6)	C108	C109	1.405(6)
C47	C48	1.377(6)	C109	C110	1.390(6)
C48	C49	1.408(6)	C109	C112	1.488(5)
C49	C50	1.404(6)	C110	C111	1.394(6)

Table 4 Bond Lengths for Li21_06.

Atom	Atom	Length/Å	Atom	Atom	Length/Å
C49	C52	1.483(6)	C113	C114	1.505(7)
C50	C51	1.383(6)	C113	C120	1.524(7)
C53	C54	1.529(6)	C114	C115	1.387(7)
C53	C60	1.521(7)	C114	C119	1.396(7)
C54	C55	1.385(6)	C115	C116	1.385(7)
C54	C59	1.383(6)	C116	C117	1.364(9)
C55	C56	1.374(7)	C117	C118	1.379(10)
C56	C57	1.380(7)	C118	C119	1.387(8)
C57	C58	1.377(7)	Cl1	C121	1.787(11)
C58	C59	1.395(7)	Cl2	C121	1.719(10)
S2	N5	1.620(4)	Cl4	C122	1.67(2)
S2	N6	1.597(4)	Cl3	C122	1.66(2)

Table 5 Bond Angles for Li21_06.

Atom	Atom	Atom	Angle/˚	Atom	Atom	Atom	Angle/˚
N1	S1	N2	102.09(19)	C62	N5	S2	105.4(3)
C2	N1	S1	105.6(3)	C63	N6	S2	105.7(3)

Table 5 Bond Angles for Li21_06.

Atom	Atom	Atom	Angle/°	Atom	Atom	Atom	Angle/°
C3	N2	S1	105.6(3)	C87	N7	C88	122.9(3)
C27	N3	C28	124.7(4)	C112	N8	C113	122.9(3)
C52	N4	C53	120.1(4)	C62	C61	C71	122.1(4)
C2	C1	C11	119.7(4)	C66	C61	C62	117.2(4)
C6	C1	C2	117.3(4)	C66	C61	C71	120.7(4)
C6	C1	C11	123.0(4)	N5	C62	C61	125.5(4)
N1	C2	C1	125.3(4)	N5	C62	C63	112.7(4)
N1	C2	C3	113.6(4)	C61	C62	C63	121.7(4)
C1	C2	C3	121.2(4)	N6	C63	C62	113.7(4)
N2	C3	C2	113.1(4)	N6	C63	C64	125.2(4)
N2	C3	C4	125.2(4)	C64	C63	C62	121.0(4)
C4	C3	C2	121.6(4)	C63	C64	C96	118.9(4)
C3	C4	C36	119.2(4)	C65	C64	C63	117.5(4)
C5	C4	C3	117.1(4)	C65	C64	C96	123.5(4)
C5	C4	C36	123.5(4)	C64	C65	C66	121.4(4)
C4	C5	C6	121.2(4)	C64	C65	C70	121.5(4)
C4	C5	C10	121.2(4)	C70	C65	C66	117.1(4)

Table 5 Bond Angles for Li21_06.

Atom	Atom	Atom	Angle/°	Atom	Atom	Atom	Angle/°
C10	C5	C6	117.6(4)	C61	C66	C65	121.1(4)
C1	C6	C5	121.6(4)	C61	C66	C67	121.9(4)
C1	C6	C7	120.9(4)	C67	C66	C65	117.0(4)
C7	C6	C5	117.4(4)	C68	C67	C66	120.3(4)
C8	C7	C6	122.3(4)	C69	C68	C67	122.8(5)
C7	C8	C9	119.3(5)	C68	C69	C70	120.9(5)
C10	C9	C8	123.9(5)	C69	C70	C65	121.9(4)
C9	C10	C5	119.5(4)	C72	C71	C61	117.7(4)
C12	C11	C1	116.4(4)	C72	C71	C76	120.1(4)
C12	C11	C16	119.5(4)	C76	C71	C61	121.5(3)
C16	C11	C1	123.9(4)	C71	C72	C73	121.3(4)
C11	C12	C13	121.7(5)	C74	C73	C72	119.8(4)
C14	C13	C12	119.8(5)	C73	C74	C75	121.0(4)
C13	C14	C15	120.7(5)	C74	C75	C76	119.8(4)
C14	C15	C16	120.4(5)	C80	C75	C74	121.1(4)
C14	C15	C20	119.2(5)	C80	C75	C76	119.1(4)
C20	C15	C16	120.3(5)	C75	C76	C71	117.5(4)

Table 5 Bond Angles for Li21_06.

Atom	Atom	Atom	Angle/˚	Atom	Atom	Atom	Angle/˚
C11	C16	C17	124.1(4)	C75	C76	C77	118.5(4)
C15	C16	C11	117.6(4)	C77	C76	C71	124.0(4)
C15	C16	C17	118.3(4)	C76	C77	C81	123.2(3)
C16	C17	C21	124.1(4)	C78	C77	C76	119.3(4)
C18	C17	C16	118.1(4)	C78	C77	C81	117.0(4)
C18	C17	C21	117.7(4)	C77	C78	C79	121.7(4)
C17	C18	C19	122.6(6)	C80	C79	C78	119.3(4)
C20	C19	C18	120.5(5)	C79	C80	C75	121.4(4)
C19	C20	C15	120.0(5)	C82	C81	C77	118.3(4)
C22	C21	C17	120.4(4)	C86	C81	C77	122.4(4)
C22	C21	C26	118.6(4)	C86	C81	C82	119.1(4)
C26	C21	C17	120.9(4)	C83	C82	C81	120.6(4)
C23	C22	C21	120.5(4)	C82	C83	C84	120.6(4)
C22	C23	C24	121.3(4)	C83	C84	C87	117.9(4)
C23	C24	C25	118.5(4)	C85	C84	C83	118.8(4)
C23	C24	C27	117.8(4)	C85	C84	C87	123.2(4)
C25	C24	C27	123.7(4)	C86	C85	C84	120.9(4)

Table 5 Bond Angles for Li21_06.

Atom	Atom	Atom	Angle/˚	Atom	Atom	Atom	Angle/˚
C26	C25	C24	120.1(4)	C85	C86	C81	119.9(4)
C25	C26	C21	121.0(4)	O3	C87	N7	123.4(4)
O1	C27	N3	121.7(4)	O3	C87	C84	121.9(4)
O1	C27	C24	120.2(4)	N7	C87	C84	114.7(3)
N3	C27	C24	118.1(4)	N7	C88	C89	113.1(4)
N3	C28	C29	111.9(4)	N7	C88	C95	108.8(4)
N3	C28	C35	109.1(4)	C89	C88	C95	111.1(3)
C35	C28	C29	115.1(4)	C90	C89	C88	118.8(4)
C30	C29	C28	121.4(4)	C94	C89	C88	123.0(4)
C30	C29	C34	117.8(5)	C94	C89	C90	118.2(4)
C34	C29	C28	120.7(5)	C91	C90	C89	121.0(5)
C29	C30	C31	120.8(5)	C92	C91	C90	120.1(5)
C32	C31	C30	120.1(5)	C93	C92	C91	119.6(5)
C33	C32	C31	119.6(5)	C92	C93	C94	120.3(5)
C32	C33	C34	121.2(5)	C89	C94	C93	120.8(5)
C29	C34	C33	120.5(6)	C97	C96	C64	117.1(4)
C37	C36	C4	115.2(4)	C97	C96	C101	119.9(4)

Table 5 Bond Angles for Li21_06.

Atom	Atom	Atom	Angle/°	Atom	Atom	Atom	Angle/°
C37	C36	C41	119.4(4)	C101	C96	C64	122.8(3)
C41	C36	C4	125.4(4)	C96	C97	C98	121.9(5)
C36	C37	C38	122.7(4)	C99	C98	C97	119.4(4)
C39	C38	C37	119.6(4)	C98	C99	C100	121.2(4)
C38	C39	C40	120.7(4)	C99	C100	C101	120.2(4)
C39	C40	C41	120.8(4)	C99	C100	C105	119.9(4)
C39	C40	C45	119.4(4)	C101	C100	C105	119.9(4)
C45	C40	C41	119.8(4)	C100	C101	C96	117.3(4)
C40	C41	C36	116.7(4)	C100	C101	C102	117.8(4)
C40	C41	C42	118.1(4)	C102	C101	C96	124.9(4)
C42	C41	C36	125.2(4)	C101	C102	C106	123.9(4)
C41	C42	C46	123.8(3)	C103	C102	C101	120.1(4)
C43	C42	C41	119.5(4)	C103	C102	C106	115.7(4)
C43	C42	C46	116.6(4)	C102	C103	C104	121.5(5)
C42	C43	C44	121.5(4)	C105	C104	C103	119.9(4)
C45	C44	C43	120.1(4)	C104	C105	C100	120.6(4)
C44	C45	C40	120.9(4)	C107	C106	C102	121.8(4)

Table 5 Bond Angles for Li21_06.

Atom	Atom	Atom	Angle/˚	Atom	Atom	Atom	Angle/˚
C47	C46	C42	120.9(4)	C111	C106	C102	119.4(4)
C51	C46	C42	120.6(4)	C111	C106	C107	118.8(4)
C51	C46	C47	118.4(4)	C108	C107	C106	120.7(4)
C48	C47	C46	120.7(4)	C107	C108	C109	120.7(4)
C47	C48	C49	121.4(4)	C108	C109	C112	121.8(4)
C48	C49	C52	119.0(4)	C110	C109	C108	118.8(4)
C50	C49	C48	117.6(4)	C110	C109	C112	119.4(4)
C50	C49	C52	123.4(4)	C109	C110	C111	119.8(4)
C51	C50	C49	120.4(4)	C106	C111	C110	121.3(4)
C50	C51	C46	121.5(4)	O4	C112	N8	123.3(4)
O2	C52	N4	119.8(4)	O4	C112	C109	121.9(4)
O2	C52	C49	120.9(4)	N8	C112	C109	114.8(3)
N4	C52	C49	119.3(4)	N8	C113	C114	112.6(3)
N4	C53	C54	110.8(3)	N8	C113	C120	108.1(4)
N4	C53	C60	109.8(4)	C114	C113	C120	110.6(4)
C60	C53	C54	111.1(4)	C115	C114	C113	122.0(4)
C55	C54	C53	120.2(4)	C115	C114	C119	118.0(4)

Table 5 Bond Angles for Li21_06.

Atom	Atom	Atom	Angle/°	Atom	Atom	Atom	Angle/°
C59	C54	C53	120.6(4)	C119	C114	C113	119.9(4)
C59	C54	C55	119.2(4)	C116	C115	C114	121.8(5)
C56	C55	C54	120.7(5)	C117	C116	C115	119.6(6)
C55	C56	C57	120.4(5)	C116	C117	C118	120.0(5)
C58	C57	C56	119.6(4)	C117	C118	C119	120.8(6)
C57	C58	C59	120.1(4)	C118	C119	C114	119.9(5)
C54	C59	C58	120.0(4)	Cl2	C121	Cl1	111.6(6)
N6	S2	N5	102.5(2)	Cl3	C122	Cl4	119.1(18)

Table 6 Torsion Angles for Li21_06.

A	B	C	D	Angle/°	A	B	C	D	Angle/°
S1	N1	C2	C1	-180.0(3)	S2	N5	C62	C61	-178.1(3)
S1	N1	C2	C3	-0.8(4)	S2	N5	C62	C63	-0.3(4)
S1	N2	C3	C2	-0.5(4)	S2	N6	C63	C62	0.3(4)
S1	N2	C3	C4	-178.8(3)	S2	N6	C63	C64	-178.3(3)

Table 6 Torsion Angles for Li21_06.

A	B	C	D	Angle/˚	A	B	C	D	Angle/˚
N1	S1	N2	C3	0.0(3)	N5	S2	N6	C63	-0.4(3)
N1	C2	C3	N2	0.9(5)	N5	C62	C63	N6	0.0(5)
N1	C2	C3	C4	179.3(4)	N5	C62	C63	C64	178.7(4)
N2	S1	N1	C2	0.5(3)	N6	S2	N5	C62	0.4(3)
N2	C3	C4	C5	-179.6(4)	N6	C63	C64	C65	-179.0(4)
N2	C3	C4	C36	-4.8(6)	N6	C63	C64	C96	-2.7(6)
N3	C28	C29	C30	-32.3(6)	N7	C88	C89	C90	-169.9(4)
N3	C28	C29	C34	151.3(5)	N7	C88	C89	C94	9.9(5)
N4	C53	C54	C55	-137.7(4)	N8	C113	C114	C115	37.2(5)
N4	C53	C54	C59	43.5(6)	N8	C113	C114	C119	-145.4(4)
C1	C2	C3	N2	-179.9(4)	C61	C62	C63	N6	177.9(4)
C1	C2	C3	C4	-1.5(6)	C61	C62	C63	C64	-3.4(6)

Table 6 Torsion Angles for Li21_06.

A	B	C	D	Angle/˚	A	B	C	D	Angle/˚
C1	C6	C7	C8	176.2(4)	C61	C66	C67	C68	-178.8(4)
C1	C11	C12	C13	171.8(5)	C61	C71	C72	C73	-168.4(4)
C1	C11	C16	C15	-168.2(4)	C61	C71	C76	C75	162.6(4)
C1	C11	C16	C17	9.7(7)	C61	C71	C76	C77	-15.5(6)
C2	C1	C6	C5	-2.0(5)	C62	C61	C66	C65	-1.3(5)
C2	C1	C6	C7	-177.6(4)	C62	C61	C66	C67	177.7(4)
C2	C1	C11	C12	-111.3(5)	C62	C61	C71	C72	-76.8(5)
C2	C1	C11	C16	63.0(6)	C62	C61	C71	C76	112.7(5)
C2	C3	C4	C5	2.2(6)	C62	C63	C64	C65	2.4(5)
C2	C3	C4	C36	177.0(4)	C62	C63	C64	C96	178.8(4)
C3	C4	C5	C6	-2.8(6)	C63	C64	C65	C66	-1.1(5)
C3	C4	C5	C10	175.3(4)	C63	C64	C65	C70	-178.2(4)

Table 6 Torsion Angles for Li21_06.

A	B	C	D	Angle/˚	A	B	C	D	Angle/˚
C3	C4	C36	C37	-69.4(5)	C63	C64	C96	C97	-94.3(5)
C3	C4	C36	C41	111.0(5)	C63	C64	C96	C101	80.3(5)
C4	C5	C6	C1	2.8(6)	C64	C65	C66	C61	0.5(5)
C4	C5	C6	C7	178.6(4)	C64	C65	C66	C67	-178.5(3)
C4	C5	C10	C9	-178.7(4)	C64	C65	C70	C69	178.6(4)
C4	C36	C37	C38	-177.0(5)	C64	C96	C97	C98	172.4(5)
C4	C36	C41	C40	176.4(4)	C64	C96	C101	C100	-170.5(4)
C4	C36	C41	C42	-2.2(7)	C64	C96	C101	C102	8.9(7)
C5	C4	C36	C37	105.1(5)	C65	C64	C96	C97	81.8(6)
C5	C4	C36	C41	-74.5(6)	C65	C64	C96	C101	-103.6(5)
C5	C6	C7	C8	0.3(7)	C65	C66	C67	C68	0.2(5)
C6	C1	C2	N1	-179.5(4)	C66	C61	C62	N5	-179.6(4)

Table 6 Torsion Angles for Li21_06.

A	B	C	D	Angle/˚	A	B	C	D	Angle/˚
C6	C1	C2	C3	1.3(5)	C66	C61	C62	C63	2.7(6)
C6	C1	C11	C12	65.3(6)	C66	C61	C71	C72	100.3(5)
C6	C1	C11	C16	-120.4(5)	C66	C61	C71	C76	-70.2(5)
C6	C5	C10	C9	-0.5(6)	C66	C65	C70	C69	1.3(6)
C6	C7	C8	C9	-1.1(8)	C66	C67	C68	C69	1.0(6)
C7	C8	C9	C10	1.1(8)	C67	C68	C69	C70	-0.9(7)
C8	C9	C10	C5	-0.2(7)	C68	C69	C70	C65	-0.2(6)
C10	C5	C6	C1	-175.3(4)	C70	C65	C66	C61	177.8(3)
C10	C5	C6	C7	0.5(6)	C70	C65	C66	C67	-1.2(5)
C11	C1	C2	N1	-2.8(6)	C71	C61	C62	N5	-2.4(6)
C11	C1	C2	C3	178.1(4)	C71	C61	C62	C63	179.9(4)
C11	C1	C6	C5	-178.7(4)	C71	C61	C66	C65	-178.5(3)
C11	C1	C6	C7	5.7(6)	C71	C61	C66	C67	0.4(6)
C11	C12	C13	C14	-2.9(8)	C71	C72	C73	C74	4.0(7)

Table 6 Torsion Angles for Li21_06.

A	B	C	D	Angle/˚	A	B	C	D	Angle/˚
C11	C16	C17	C18	-171.4(5)	C71	C76	C77	C78	170.6(4)
C11	C16	C17	C21	13.4(7)	C71	C76	C77	C81	-17.6(6)
C12	C11	C16	C15	5.9(7)	C72	C71	C76	C75	-7.7(6)
C12	C11	C16	C17	-176.3(5)	C72	C71	C76	C77	174.2(4)
C12	C13	C14	C15	5.0(8)	C72	C73	C74	C75	-4.6(7)
C13	C14	C15	C16	-1.6(8)	C73	C74	C75	C76	-1.1(7)
C13	C14	C15	C20	-179.1(5)	C73	C74	C75	C80	177.2(4)
C14	C15	C16	C11	-3.8(7)	C74	C75	C76	C71	7.1(6)
C14	C15	C16	C17	178.2(5)	C74	C75	C76	C77	-174.7(4)
C14	C15	C20	C19	176.6(5)	C74	C75	C80	C79	-179.5(4)
C15	C16	C17	C18	6.5(7)	C75	C76	C77	C78	-7.5(6)
C15	C16	C17	C21	-168.7(4)	C75	C76	C77	C81	164.3(4)

Table 6 Torsion Angles for Li21_06.

A	B	C	D	Angle/°	A	B	C	D	Angle/°
C16	C11	C12	C13	-2.7(7)	C76	C71	C72	C73	2.2(7)
C16	C15	C20	C19	-0.9(8)	C76	C75	C80	C79	-1.2(6)
C16	C17	C18	C19	-3.7(9)	C76	C77	C78	C79	2.2(6)
C16	C17	C21	C22	-126.5(5)	C76	C77	C81	C82	-65.4(5)
C16	C17	C21	C26	58.0(6)	C76	C77	C81	C86	120.3(4)
C17	C18	C19	C20	-1.6(10)	C77	C78	C79	C80	3.7(7)
C17	C21	C22	C23	-177.0(4)	C77	C81	C82	C83	-173.5(4)
C17	C21	C26	C25	177.3(4)	C77	C81	C86	C85	172.4(3)
C18	C17	C21	C22	58.2(6)	C78	C77	C81	C82	106.6(4)
C18	C17	C21	C26	-117.2(5)	C78	C77	C81	C86	-67.7(5)
C18	C19	C20	C15	4.0(9)	C78	C79	C80	C75	-4.1(7)
C20	C15	C16	C11	173.7(5)	C80	C75	C76	C71	-171.2(4)
C20	C15	C16	C17	-4.3(7)	C80	C75	C76	C77	7.0(6)

Table 6 Torsion Angles for Li21_06.

A	B	C	D	Angle/˚	A	B	C	D	Angle/˚
C21	C17	C18	C19	171.8(5)	C81	C77	C78	C79	-170.1(4)
C21	C22	C23	C24	0.2(6)	C81	C82	C83	C84	0.4(6)
C22	C21	C26	C25	1.8(6)	C82	C81	C86	C85	-1.9(6)
C22	C23	C24	C25	0.8(6)	C82	C83	C84	C85	-0.9(6)
C22	C23	C24	C27	179.4(4)	C82	C83	C84	C87	175.7(4)
C23	C24	C25	C26	-0.5(6)	C83	C84	C85	C86	0.0(6)
C23	C24	C27	O1	10.6(6)	C83	C84	C87	O3	43.3(6)
C23	C24	C27	N3	-168.3(4)	C83	C84	C87	N7	-133.8(4)
C24	C25	C26	C21	-0.8(6)	C84	C85	C86	C81	1.4(6)
C25	C24	C27	O1	-170.8(4)	C85	C84	C87	O3	-140.3(4)
C25	C24	C27	N3	10.2(6)	C85	C84	C87	N7	42.6(5)
C26	C21	C22	C23	-1.5(6)	C86	C81	C82	C83	1.0(6)
C27	N3	C28	C29	-58.9(6)	C87	N7	C88	C89	90.9(5)
C27	N3	C28	C35	69.7(5)	C87	N7	C88	C95	-145.1(4)

Table 6 Torsion Angles for Li21_06.

A	B	C	D	Angle/˚	A	B	C	D	Angle/˚
C27	C24	C25	C26	-179.0(4)	C87	C84	C85	C86	-176.4(4)
C28	N3	C27	O1	7.4(6)	C88	N7	C87	O3	-1.4(7)
C28	N3	C27	C24	-173.7(4)	C88	N7	C87	C84	175.7(4)
C28	C29	C30	C31	-178.8(4)	C88	C89	C90	C91	179.1(4)
C28	C29	C34	C33	177.5(5)	C88	C89	C94	C93	-178.8(4)
C29	C30	C31	C32	1.4(7)	C89	C90	C91	C92	0.0(7)
C30	C29	C34	C33	1.0(7)	C90	C89	C94	C93	1.0(6)
C30	C31	C32	C33	0.9(8)	C90	C91	C92	C93	0.2(8)
C31	C32	C33	C34	-2.2(9)	C91	C92	C93	C94	0.1(8)
C32	C33	C34	C29	1.2(8)	C92	C93	C94	C89	-0.8(7)
C34	C29	C30	C31	-2.3(7)	C94	C89	C90	C91	-0.6(7)
C35	C28	C29	C30	-157.7(5)	C95	C88	C89	C90	67.5(5)

Table 6 Torsion Angles for Li21_06.

A	B	C	D	Angle/˚	A	B	C	D	Angle/˚
C35	C28	C29	C34	26.0(7)	C95	C88	C89	C94	-112.8(5)
C36	C4	C5	C6	-177.4(4)	C96	C64	C65	C66	-177.2(4)
C36	C4	C5	C10	0.7(6)	C96	C64	C65	C70	5.6(6)
C36	C37	C38	C39	-0.4(8)	C96	C97	C98	C99	0.0(9)
C36	C41	C42	C43	175.6(4)	C96	C101	C102	C103	-174.3(4)
C36	C41	C42	C46	-7.3(7)	C96	C101	C102	C106	12.1(7)
C37	C36	C41	C40	-3.3(6)	C97	C96	C101	C100	3.9(7)
C37	C36	C41	C42	178.2(4)	C97	C96	C101	C102	-176.7(5)
C37	C38	C39	C40	-1.2(8)	C97	C98	C99	C100	0.6(9)
C38	C39	C40	C41	0.4(7)	C98	C99	C100	C101	1.2(8)
C38	C39	C40	C45	-179.7(5)	C98	C99	C100	C105	-179.0(5)
C39	C40	C41	C36	1.8(7)	C99	C100	C101	C96	-3.4(7)

Table 6 Torsion Angles for Li21_06.

A	B	C	D	Angle/˚	A	B	C	D	Angle/˚
C39	C40	C41	C42	-179.5(4)	C99	C100	C101	C102	177.2(4)
C39	C40	C45	C44	-178.0(5)	C99	C100	C105	C104	178.8(5)
C40	C41	C42	C43	-2.9(7)	C100	C101	C102	C103	5.1(7)
C40	C41	C42	C46	174.2(4)	C100	C101	C102	C106	-168.5(4)
C41	C36	C37	C38	2.6(7)	C101	C96	C97	C98	-2.3(8)
C41	C40	C45	C44	2.0(7)	C101	C100	C105	C104	-1.3(8)
C41	C42	C43	C44	2.9(7)	C101	C102	C103	C104	-3.7(7)
C41	C42	C46	C47	116.9(5)	C101	C102	C106	C107	-103.0(5)
C41	C42	C46	C51	-66.5(6)	C101	C102	C106	C111	80.3(5)
C42	C43	C44	C45	-0.5(8)	C102	C103	C104	C105	-0.4(8)
C42	C46	C47	C48	179.7(4)	C102	C106	C107	C108	-178.0(4)
C42	C46	C51	C50	-179.8(4)	C102	C106	C111	C110	178.8(4)

Table 6 Torsion Angles for Li21_06.

A	B	C	D	Angle/˚	A	B	C	D	Angle/˚
C43	C42	C46	C47	-65.9(6)	C103	C102	C106	C107	83.1(5)
C43	C42	C46	C51	110.7(5)	C103	C102	C106	C111	-93.6(5)
C43	C44	C45	C40	-2.0(8)	C103	C104	C105	C100	2.9(8)
C45	C40	C41	C36	-178.1(4)	C105	C100	C101	C96	176.8(4)
C45	C40	C41	C42	0.5(7)	C105	C100	C101	C102	-2.7(7)
C46	C42	C43	C44	-174.4(4)	C106	C102	C103	C104	170.4(5)
C46	C47	C48	C49	-0.9(7)	C106	C107	C108	C109	0.0(6)
C47	C46	C51	C50	-3.1(6)	C107	C106	C111	C110	1.9(6)
C47	C48	C49	C50	-1.1(6)	C107	C108	C109	C110	0.5(5)
C47	C48	C49	C52	178.5(4)	C107	C108	C109	C112	-179.3(3)
C48	C49	C50	C51	1.0(6)	C108	C109	C110	C111	0.2(5)
C48	C49	C52	O2	-5.1(6)	C108	C109	C112	O4	146.7(4)
C48	C49	C52	N4	174.4(4)	C108	C109	C112	N8	-32.4(5)
C49	C50	C51	C46	1.2(6)	C109	C110	C111	C106	-1.5(6)

Table 6 Torsion Angles for Li21_06.

A	B	C	D	Angle/°	A	B	C	D	Angle/°
C50	C49	C52	O2	174.5(4)	C110	C109	C112	O4	-33.2(5)
C50	C49	C52	N4	-6.0(6)	C110	C109	C112	N8	147.8(4)
C51	C46	C47	C48	3.0(6)	C111	C106	C107	C108	-1.2(6)
C52	N4	C53	C54	72.5(5)	C112	N8	C113	C114	87.1(5)
C52	N4	C53	C60	-164.4(4)	C112	N8	C113	C120	-150.4(4)
C52	C49	C50	C51	-178.6(4)	C112	C109	C110	C111	-179.9(3)
C53	N4	C52	O2	3.3(6)	C113	N8	C112	O4	1.3(6)
C53	N4	C52	C49	-176.3(4)	C113	N8	C112	C109	-179.7(4)
C53	C54	C55	C56	-179.5(4)	C113	C114	C115	C116	178.0(4)
C53	C54	C59	C58	179.6(4)	C113	C114	C119	C118	-178.0(4)
C54	C55	C56	C57	0.1(8)	C114	C115	C116	C117	0.2(8)
C55	C54	C59	C58	0.7(7)	C115	C114	C119	C118	-0.5(7)
C55	C56	C57	C58	0.5(8)	C115	C116	C117	C118	-1.1(8)

Table 6 Torsion Angles for Li21_06.

A	B	C	D	Angle/°	A	B	C	D	Angle/°
C56	C57	C58	C59	-0.5(8)	C116	C117	C118	C119	1.1(8)
C57	C58	C59	C54	-0.1(7)	C117	C118	C119	C114	-0.3(8)
C59	C54	C55	C56	-0.7(7)	C119	C114	C115	C116	0.5(7)
C60	C53	C54	C55	100.0(5)	C120	C113	C114	C115	-83.9(5)
C60	C53	C54	C59	-78.8(5)	C120	C113	C114	C119	93.5(5)

Table 7 Hydrogen Atom Coordinates (Å×10^4) and Isotropic Displacement Parameters (Å^2×10^3) for Li21_06.

Atom	*x*	*y*	*z*	U(eq)
H3	5821.76	2183	-540.34	48
H4	8607.77	5330.68	4898.18	47
H7	5279.14	5094.44	1957.89	51
H8	3108.17	4200.14	1595.99	63
H9	2823.61	2579.24	1573.52	51
H10	4627.97	1780.46	1939.15	51
H12	6807.95	5681.16	3237.88	59
H13	7287.04	7340.45	3456.22	70

Table 7 Hydrogen Atom Coordinates (Å×10^4) and Isotropic Displacement Parameters (Å^2×10^3) for Li21_06.

Atom	*x*	*y*	*z*	U(eq)
H14	8868.95	8254.38	2838.19	67
H18	10718.65	6148.12	632.86	71
H19	11372.06	7756.59	994.86	88
H20	10321.76	8428.27	1875.42	78
H22	10808.01	4439.96	1136.87	48
H23	10094.84	2935.83	560.64	46
H25	6085.25	3378.42	148.13	45
H26	6810.8	4898.73	719.44	43
H28	4854.7	819.29	-1105.27	55
H30	7341.31	1784.98	-1719.46	55
H31	8683.88	1286.01	-2565.96	67
H32	8789.39	-318.6	-2726.36	76
H33	7533.24	-1413.33	-2078.27	83
H34	6256.7	-935.66	-1197.43	70
H35A	4960.68	-476.73	-403.57	95
H35B	5074.63	411.11	170.67	95

Table 7 Hydrogen Atom Coordinates (Å×10[4]) and Isotropic Displacement Parameters (Å[2]×10[3]) for Li21_06.

Atom	*x*	*y*	*z*	U(eq)
H35C	6448.55	-8.32	70.46	95
H37	8123.7	1468.43	1480.52	53
H38	7949.73	-160	1278.92	58
H39	6899.28	-1185.45	2062.8	54
H43	5002.73	683.76	4393.82	54
H44	4856.47	-950.64	4139.35	61
H45	5798.7	-1479.97	3161.71	59
H47	4114.28	2235.02	3646.83	47
H48	4362.53	3810.09	4046.69	46
H50	8585	3972.25	4518.08	44
H51	8309.4	2389.66	4118.61	41
H53	7406.78	6907.36	4752.65	50
H55	6316.76	7702.23	5592.51	52
H56	5393.23	7774.37	6686.56	60
H57	5853.21	6725.05	7621.22	59
H58	7274.12	5613.03	7460.14	59

Table 7 Hydrogen Atom Coordinates (Å×10[4]) and Isotropic Displacement Parameters (Å[2]×10[3]) for Li21_06.

Atom	*x*	*y*	*z*	U(eq)
H59	8212.21	5537.45	6354.26	53
H60A	10053.89	6700.39	5593.35	90
H60B	9556.08	7704.47	5485.46	90
H60C	9808.07	7107.76	4776.73	90
H7A	3622.36	5610.84	4453.29	44
H8A	432.18	1321.82	9640.03	40
H67	5002.69	2573	7992.06	42
H68	6669.66	3723.56	8676.44	55
H69	6307.05	5220.34	8860.32	59
H70	4271.71	5675.9	8344.63	44
H72	1419.11	1247.34	7929.76	50
H73	1976.12	-286.2	7988.5	56
H74	3742.61	-655.37	7401.96	54
H78	5708.5	2246.48	5550.47	49
H79	6598.78	849.7	5844.72	52
H80	5503.67	-218.63	6586.57	53

Table 7 Hydrogen Atom Coordinates (Å×10^4) and Isotropic Displacement Parameters (Å^2×10^3) for Li21_06.

Atom	*x*	*y*	*z*	U(eq)
H82	1764.54	2099.7	5411.98	45
H83	816.3	3315.28	4817.93	44
H85	4312.16	5183.08	5682.45	38
H86	5248.04	3971.05	6307.63	41
H88	1293.59	6178.19	3660.39	43
H90	1389.52	7936.58	3388.22	54
H91	1521.22	9431.27	3961.99	64
H92	2644.84	9764.54	5199.05	61
H93	3631.84	8609.85	5849.84	64
H94	3522.48	7103	5268.61	54
H95A	4154.21	6633.59	3442.78	76
H95B	2867.01	6931.68	2895.77	76
H95C	2987.22	5834.71	2965.41	76
H97	2851.9	6081.73	6739.22	57
H98	2313.3	7619.73	6658.8	68
H99	910.13	8118.89	7433.54	64

Table 7 Hydrogen Atom Coordinates (Å×10^4) and Isotropic Displacement Parameters (Å^2×10^3) for Li21_06.

Atom	*x*	*y*	*z*	U(eq)
H103	-846.8	5352.51	9429.91	54
H104	-1450.27	6861.46	9273.59	63
H105	-498.72	7846.59	8430.99	61
H107	-773.62	3588.22	8335.29	41
H108	-28.32	2210.05	8747.72	38
H110	3501.03	3772.73	9948.28	40
H111	2706.02	5154.96	9545.63	41
H113	2461.36	197.99	9819.14	45
H115	438.95	1117.59	11051.24	54
H116	784.88	1032.05	12346.28	74
H117	2425.88	182	12929.04	78
H118	3669.43	-624.89	12217.16	83
H119	3339.74	-545.75	10918.22	62
H12A	-486.09	-305.63	9822.27	77
H12B	548.31	-1059.14	9828.53	77
H12C	263.55	-438.51	9114.87	77

Table 7 Hydrogen Atom Coordinates (Å×10[4]) and Isotropic Displacement Parameters (Å[2]×10[3]) for Li21_06.

Atom	*x*	*y*	*z*	U(eq)
H12D	9367.6	9380.87	4459.36	86
H12E	8040.43	9908.23	4371.46	86
H12F	9789.81	9207.31	4849.98	148
H12G	8210.82	9229.92	4483.45	148

Table 8 Atomic Occupancy for Li21_06.

Atom	*Occupancy*	Atom	*Occupancy*	Atom	*Occupancy*
Cl1	0.686(4)	Cl2	0.686(4)	C121	0.686(4)
H12D	0.686(4)	H12E	0.686(4)	Cl4	0.314(4)
Cl3	0.314(4)	C122	0.314(4)	H12F	0.314(4)
H12G	0.314(4)				
